# Supplementary material for: CRISPR/Cas9-mediated targeted mutagenesis of GmTCP19L increasing susceptibility to Phytophthora sojae in soybean
Source: PLoS One. 2022 Jun 9;17(6):e0267502. doi: 10.1371/journal.pone.0267502 (PMC9182224; doi:10.1371/journal.pone.0267502)
Supplement: S2 Fig — GmU6, Glycine max U6 promoter. sgRNA, small guide-RNA. GmTCP19L-SP1/SP2, two target sites in the exon of GmTCP19L. Cas9-F/R, the primers of the detection region for Cas9. NLS, nuclear localization sequence. The bar gene driven by a CaMV 35S promoter is used as a screening marker. Kan, kanamycin resistance gene. pVS1, pVS1 replication origin. STA, pVS1 stability function. (PDF) [file pone.0267502.s002.pdf]

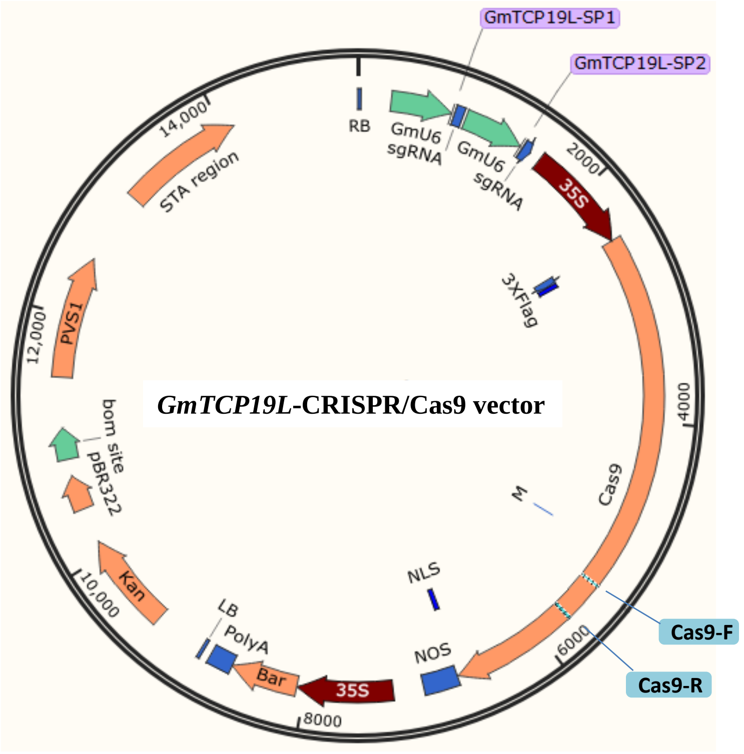

S2 Fig. The basic architecture of the constructs used for *GmTCP19L*-CRISPR/Cas9-mediated genome editing. GmU6, *Glycine max U6* promoter. sgRNA, small guide-RNA. *GmTCP19L*-SP1/SP2, two target sites in the exon of *GmTCP19L*. Cas9-F/R, the primers of the detection region for Cas9. NLS, nuclear localization sequence. The *bar* gene driven by a CaMV 35S promoter is used as a screening marker. Kan, kanamycin resistance gene. pVS1, pVS1 replication origin. STA, pVS1 stability function.
