# Supplementary material for: CRISPR/Cas9-mediated targeted mutagenesis of GmTCP19L increasing susceptibility to Phytophthora sojae in soybean
Source: PLoS One. 2022 Jun 9;17(6):e0267502. doi: 10.1371/journal.pone.0267502 (PMC9182224; doi:10.1371/journal.pone.0267502)
Supplement: S3 Fig — (A) The PCR amplified products of GmTCP19L. M, DL2000 DNA Marker. (B) Phylogenetic tree analysis of GmTCP19L with the 24 TCP transcription factor members of Arabidopsis. (PDF) [file pone.0267502.s003.pdf]

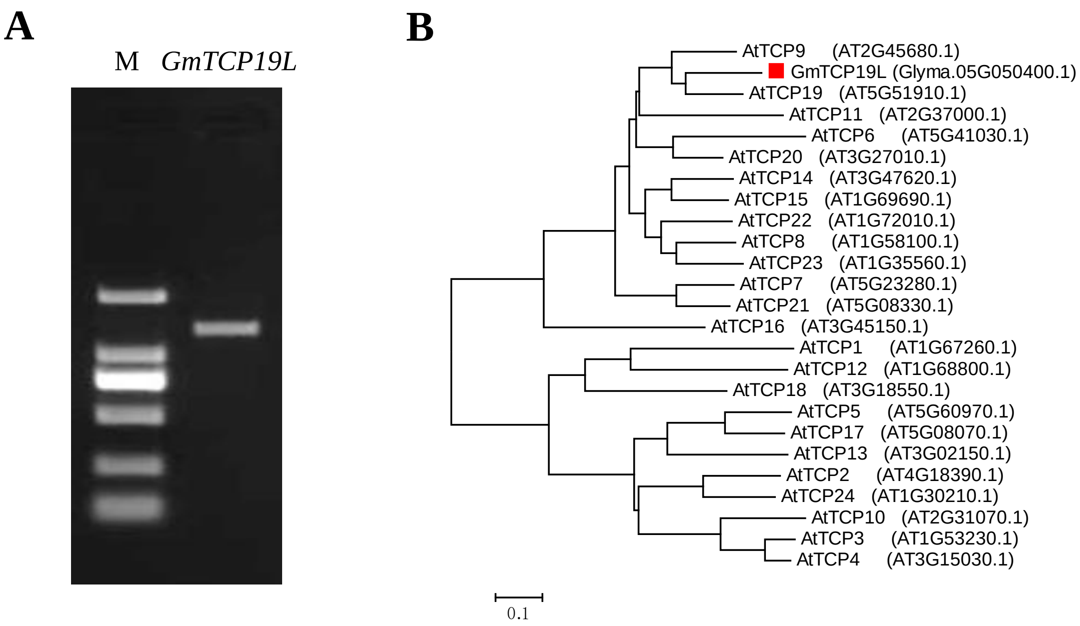

S3 Fig. Phylogenetic tree analysis of GmTCP19L with the 24 TCP transcription factor members of Arabidopsis. (A) The PCR amplified products of *GmTCP19L*. M, DL2000 DNA Maker. **(B)** Phylogenetic tree analysis of GmTCP19L with the 24 TCP transcription factor members of Arabidopsis.
