# Supplementary material for: CRISPR/Cas9-mediated targeted mutagenesis of GmTCP19L increasing susceptibility to Phytophthora sojae in soybean
Source: PLoS One. 2022 Jun 9;17(6):e0267502. doi: 10.1371/journal.pone.0267502 (PMC9182224; doi:10.1371/journal.pone.0267502)
Supplement: S4 Fig — WT, wild-type soybean plant. (PDF) [file pone.0267502.s004.pdf]

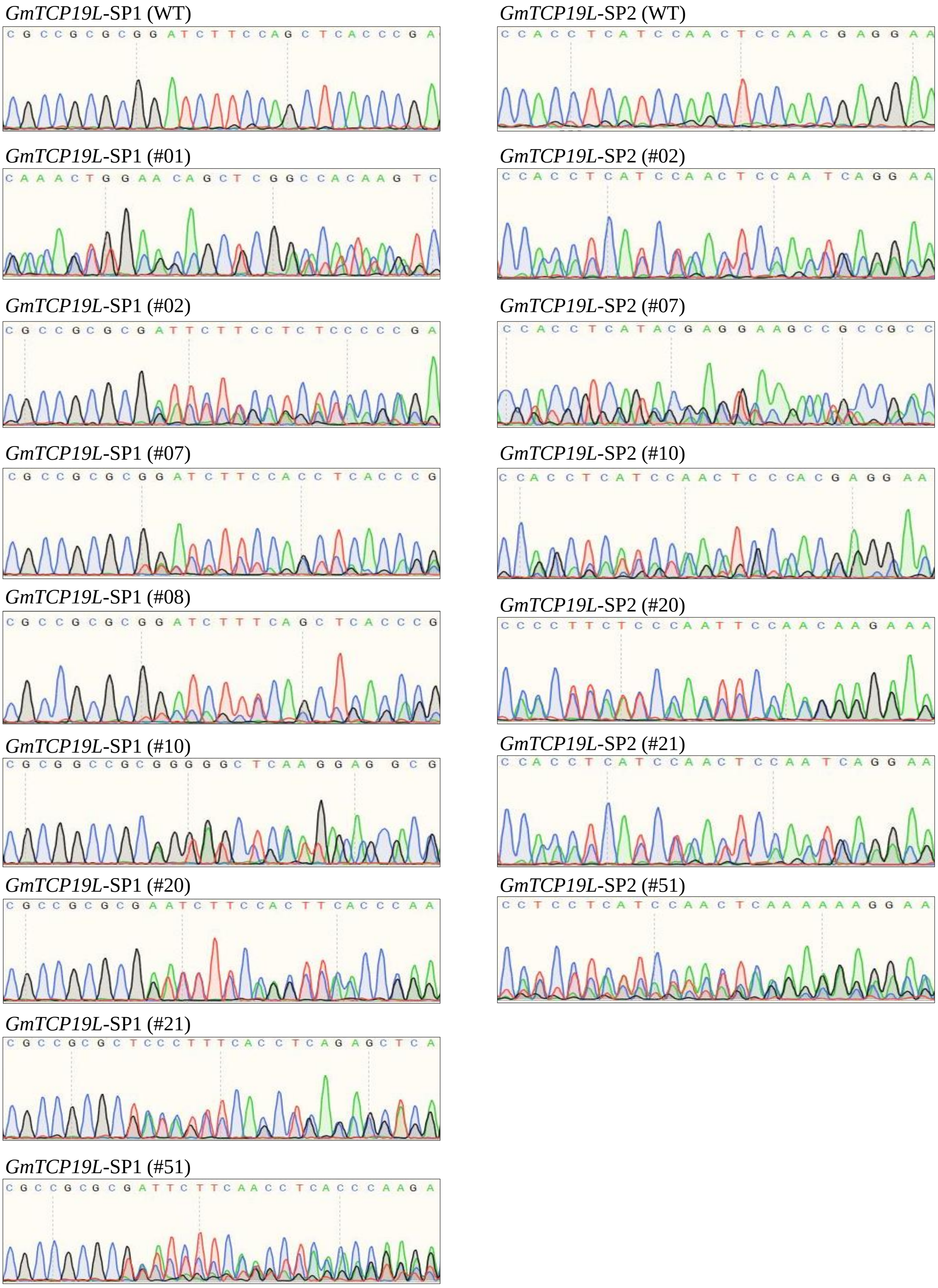

S4 Fig. Heterozygous targeted mutagenesis of *GmTCP19L* induced by CRISPR/Cas9 in the T0 generation. WT, wild-type soybean plant.
