# Supplementary material for: CRISPR/Cas9-mediated targeted mutagenesis of GmTCP19L increasing susceptibility to Phytophthora sojae in soybean
Source: PLoS One. 2022 Jun 9;17(6):e0267502. doi: 10.1371/journal.pone.0267502 (PMC9182224; doi:10.1371/journal.pone.0267502)
Supplement: S5 Fig — The TCP-like domain was marked with a black box. Nuclear location signal was marked with a dashed black box. (PDF) [file pone.0267502.s005.pdf]

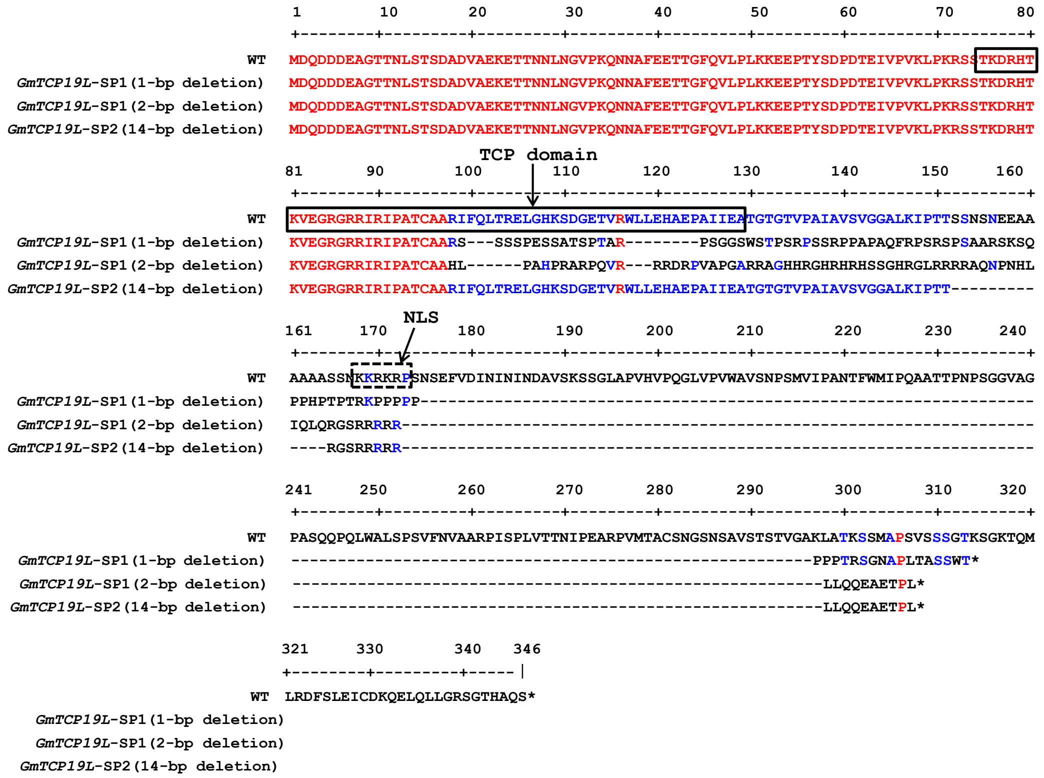

S5 Fig. Amino acid sequence alignment of *GmTCP19L* mutations with WT. The TCP-like domain was marked with a black box. Nuclear location signal was marked with a dashed black box.
