# Supplementary material for: CRISPR/Cas9-mediated targeted mutagenesis of GmTCP19L increasing susceptibility to Phytophthora sojae in soybean
Source: PLoS One. 2022 Jun 9;17(6):e0267502. doi: 10.1371/journal.pone.0267502 (PMC9182224; doi:10.1371/journal.pone.0267502)
Supplement: S6 Fig — CDS, coding sequence. Blue capital letter, target sequence. Red capital letter, protospacer adjacent motif. Dashes, deletions. Yellow rectangle, termination codon. (PDF) [file pone.0267502.s006.pdf]

CDS of *GmTCP19L* (WT, wild-type)
ATGGATCAAGACGACGACGAAGCAGGAACAACGAACCTCAGCACCAGCGACGCCGACGTGGCAGA
GAAAGAAACCACAAACAACCTCAATGGAGTCCCCAAACAGAACAACGCGTTTGAAGAAACAACAGG
TTTTCAAGTCTTGCCGCTGAAAAAGGAGGAGCCAACATATTCGGACCCTGACACGGAGATTGTTCCG
GTCAAACTGCCAAAGCGCTCCTCCACGAAGGACCGCCACACCAAAGTGGAAGGGCGCGGCCGCAGG
ATCCGAATACCCGCCACCTGCGCCGCGCGGATCTTCCAGCTCACCCGAGAGCTCGGCCACAAGTCCG
ACGGCGAGACCGTCCGGTGGCTCCTGGAGCACGCCGAGCCGGCCATCATCGAGGCCACCGGCACCG
GCACAGTTCCGGCCATCGCGGTCTCCGTCGGCGGCGCGCTCAAAATCCCAACCACCTCATCCAACTC
CAACGAGGAAGCCGCCGCCGCCGCCGCCTCCTCCAACAAGAAGCGGAAACGCCCCTCTAACAGCGA
GTTCGTGGACATAAACATAAACATAAACGACGCCGTTTCAAAGTCGTCCGGTCTGGCCCCGGTTCAT
GTGCCGCAGGGCCTGGTCCCGGTCTGGGCCGTAAGTAACCCCAGCATGGTGATCCCCGCTAACACTT
TCTGGATGATCCCTCAGGCCGCGACCACACCTAACCCCTCGGGTGGAGTAGCGGGCCCTGCAAGCCA
GCAGCCTCAGTTATGGGCCTTATCTCCCTCCGTGTTCAACGTCGCCGCCAGGCCCATATCGCCTCTGG
TTACAACCAATATTCCAGAAGCCCGGCCCGTTATGACGGCCTGCAGTAACGGTTCCAACTCCGCCGT
TAGCACGAGCACGGTGGGGGCTAAGCTGGCAACGAAATCCAGCATGGCACCCAGTGTTAGTTCTAGC
GGAACCAAGAGTGGCAAGACTCAAATGTTAAGGGATTTTTCGCTTGAAATTTGCGATAAACAAGAGT
TACAGCTCTTGGGCCGCTCTGGGACTCACGCGCAGAGTTAG

***tcp19l*-SP1-T1#02.03 (2-bp deletion)**
ATGGATCAAGACGACGACGAAGCAGGAACAACGAACCTCAGCACCAGCGACGCCGACGTGGCAGA
GAAAGAAACCACAAACAACCTCAATGGAGTCCCCAAACAGAACAACGCGTTTGAAGAAACAACAGG
TTTTCAAGTCTTGCCGCTGAAAAAGGAGGAGCCAACATATTCGGACCCTGACACGGAGATTGTTCCG
GTCAAACTGCCAAAGCGCTCCTCCACGAAGGACCGCCACACCAAAGTGGAAGGGCGCGGCCGCAGG
ATCCGAATACCCGCCACCTGCGCCGCGC--ATCTTCCAGCTCACCCGAGAGCTCGGCCACAAGTCCGA
CGGCGAGACCGTCCGGTGGCTCCTGGAGCACGCCGAGCCGGCCATCATCGAGGCCACCGGCACCGG
CACAGTTCCGGCCATCGCGGTCTCCGTCGGCGGCGCGCTCAAAATCCCAACCACCTCATCCAACTCC
AACGAGGAAGCCGCCGCCGCCGCCGCCTCCTCCAACAAGAAGCGGAAACGCCCCTCTAACAGCGAG
TTCGTGGACATAAACATAAACATAAACGACGCCGTTTCAAAGTCGTCCGGTCTGGCCCCGGTTCATG
TGCCGCAGGGCCTGGTCCCGGTCTGGGCCGTAAGTAACCCCAGCATGGTGATCCCCGCTAACACTTT
CTGGATGATCCCTCAGGCCGCGACCACACCTAACCCCTCGGGTGGAGTAGCGGGCCCTGCAAGCCAG
CAGCCTCAGTTATGGGCCTTATCTCCCTCCGTGTTCAACGTCGCCGCCAGGCCCATATCGCCTCTGGT
TACAACCAATATTCCAGAAGCCCGGCCCGTTATGACGGCCTGCAGTAACGGTTCCAACTCCGCCGTT
AGCACGAGCACGGTGGGGGCTAAGCTGGCAACGAAATCCAGCATGGCACCCAGTGTTAGTTCTAGC
GGAACCAAGAGTGGCAAGACTCAAATGTTAAGGGATTTTTCGCTTGAAATTTGCGATAAACAAGAGT
TACAGCTCTTGGGCCGCTCTGGGACTCACGCGCAGAGTTAG

***tcp19l*-SP1-T1#02.08 (2-bp deletion)**
ATGGATCAAGACGACGACGAAGCAGGAACAACGAACCTCAGCACCAGCGACGCCGACGTGGCAGA
GAAAGAAACCACAAACAACCTCAATGGAGTCCCCAAACAGAACAACGCGTTTGAAGAAACAACAGG
TTTTCAAGTCTTGCCGCTGAAAAAGGAGGAGCCAACATATTCGGACCCTGACACGGAGATTGTTCCG
GTCAAACTGCCAAAGCGCTCCTCCACGAAGGACCGCCACACCAAAGTGGAAGGGCGCGGCCGCAGG
ATCCGAATACCCGCCACCTGCGCCGCGC--ATCTTCCAGCTCACCCGAGAGCTCGGCCACAAGTCCGA
CGGCGAGACCGTCCGGTGGCTCCTGGAGCACGCCGAGCCGGCCATCATCGAGGCCACCGGCACCGG
CACAGTTCCGGCCATCGCGGTCTCCGTCGGCGGCGCGCTCAAAATCCCAACCACCTCATCCAACTCC
AACGAGGAAGCCGCCGCCGCCGCCGCCTCCTCCAACAAGAAGCGGAAACGCCCCTCTAACAGCGAG
TTCGTGGACATAAACATAAACATAAACGACGCCGTTTCAAAGTCGTCCGGTCTGGCCCCGGTTCATG
TGCCGCAGGGCCTGGTCCCGGTCTGGGCCGTAAGTAACCCCAGCATGGTGATCCCCGCTAACACTTT
CTGGATGATCCCTCAGGCCGCGACCACACCTAACCCCTCGGGTGGAGTAGCGGGCCCTGCAAGCCAG
CAGCCTCAGTTATGGGCCTTATCTCCCTCCGTGTTCAACGTCGCCGCCAGGCCCATATCGCCTCTGGT
TACAACCAATATTCCAGAAGCCCGGCCCGTTATGACGGCCTGCAGTAACGGTTCCAACTCCGCCGTT
AGCACGAGCACGGTGGGGGCTAAGCTGGCAACGAAATCCAGCATGGCACCCAGTGTTAGTTCTAGC
GGAACCAAGAGTGGCAAGACTCAAATGTTAAGGGATTTTTCGCTTGAAATTTGCGATAAACAAGAGT
TACAGCTCTTGGGCCGCTCTGGGACTCACGCGCAGAGTTAG

S6 Fig. Frameshift mutations at two target sites of *GmTCP19L* generated premature translation termination codons. CDS, coding sequence. Blue capital letter, target sequence. Red capital letter, protospacer adjacent motif. Dashes, deletions. Yellow rectangle, termination codon.
