# Supplementary material for: CRISPR/Cas9-mediated targeted mutagenesis of GmTCP19L increasing susceptibility to Phytophthora sojae in soybean
Source: PLoS One. 2022 Jun 9;17(6):e0267502. doi: 10.1371/journal.pone.0267502 (PMC9182224; doi:10.1371/journal.pone.0267502)
Supplement: S1 Table — (PDF) [file pone.0267502.s007.pdf]

S1 Table. Primer sequences used in the present study.

Primer name	Primer sequence	Purpose
TCP19L-F	ATGGATCAAGACGACGACGA	To amplify the CDS regions of *GmTCP19L*
TCP19L-R	ACTCTGCGCGTGAGTCCC	
GmTCP19L-F	GTCCCCAAACAGAACAACGC	To amplify the regions which span the target site
GmTCP19L-R	TACTCCACCCGAGGGGTTAG	
OFF1-F	GGCGTTTCCGCTTCTTGT	To examine potential off-target site 1
OFF1-R	AACCGACCTCAGCACCAG	
OFF2-F	CATAATGAGCCGAAAACTGC	To examine potential off-target site 2
OFF2-R	TGGAAAACCCATTGAAACC	
OFF3-F	TGGCTTTTGTTTGAGGGTG	To examine potential off-target site 3
OFF3-R	GGCTCTTGACTTTTCTTAGGCT	
OFF4-F	CCAAAATCAAGGACCAAAAC	To examine potential off-target site 4
OFF4-R	AAATGTAATCCATCAAGACCAAG	
Cas9-F	CTCCCGGATGAACACTAAGTAC	To amplify a part of the Cas9 coding sequence
Cas9-R	CAGGGTAATCTCGGTCTTGAAA	
PsACT-F	ACTGCACCTTCCAGACCATC	*P. sojae actin* gene used for qPCR measurement of pathogen levels
PsACT-R	CCACCACCTTGATCTTCATG	
GmCYP2-F	CCCCTCCACTACAAAGGCTCG	*G. max CYP2* gene used for qPCR measurement of pathogen levels
GmCYP2-R	CGGGACCAGTGTGCTTCTTCA	
GmActin-F	CGGTGGTTCTATCTTGGCATC	as qRT-PCR reference
GmActin-R	GTCTTTCGCTTCAATAACCCTA	
