# Supplementary material for: CRISPR/Cas9-mediated targeted mutagenesis of GmTCP19L increasing susceptibility to Phytophthora sojae in soybean
Source: PLoS One. 2022 Jun 9;17(6):e0267502. doi: 10.1371/journal.pone.0267502 (PMC9182224; doi:10.1371/journal.pone.0267502)
Supplement: S2 Table — (PDF) [file pone.0267502.s008.pdf]

S2 Table. Potential off-target analysis at the two target sites of *GmTCP19L* in the T1 generation.

Target site	Putative off-target site				No. of plans sequenced[c]	No. of plants with mutations
	Gene locus	Region	Sequence[a]	MMs[b]		
GmTCP19L-SP1	Glyma17g14160 17:-10922684	exon	GGTcAGCTGGAAGATCCGgGCGG	2	29	0
	Glyma10g17850 10:-22202782	exon	GGgGcGCTtGAAGATCCGCaAGG	4	29	0
GmTCP19L-SP2	Glyma04g15120 4:-15495238	exon	CACCTCAgCaAACTCCAACtGGG	3	15	0
	Glyma05g24520 5:-30684999	intron	aACCTCATCCAAgTCCAAgGTAG	3	15	0

a Mismatched bases are shown in lowercase letters.

b No. of mismatched bases.

c T1 plants identified as biallelic mutants of *GmTCP19L*.
