# Supplementary material for: CRISPR/Cas9-mediated targeted mutagenesis of GmTCP19L increasing susceptibility to Phytophthora sojae in soybean
Source: PLoS One. 2022 Jun 9;17(6):e0267502. doi: 10.1371/journal.pone.0267502 (PMC9182224; doi:10.1371/journal.pone.0267502)
Supplement: S1 Raw images — (PDF) [file pone.0267502.s009.pdf]

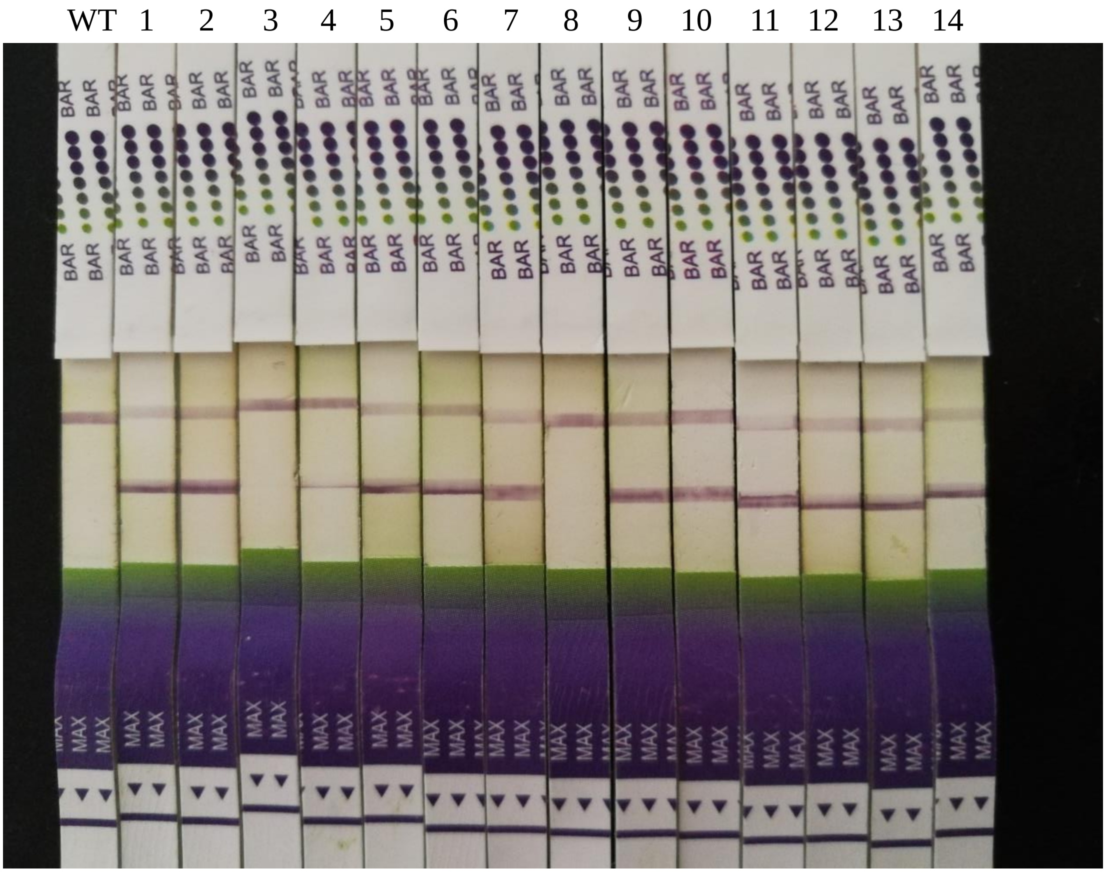

Fig 4. (A) Detection of the selectable marker gene *bar* by test strip. WT, wild type soybean plant. Labels 1–14, individual mutant plants.

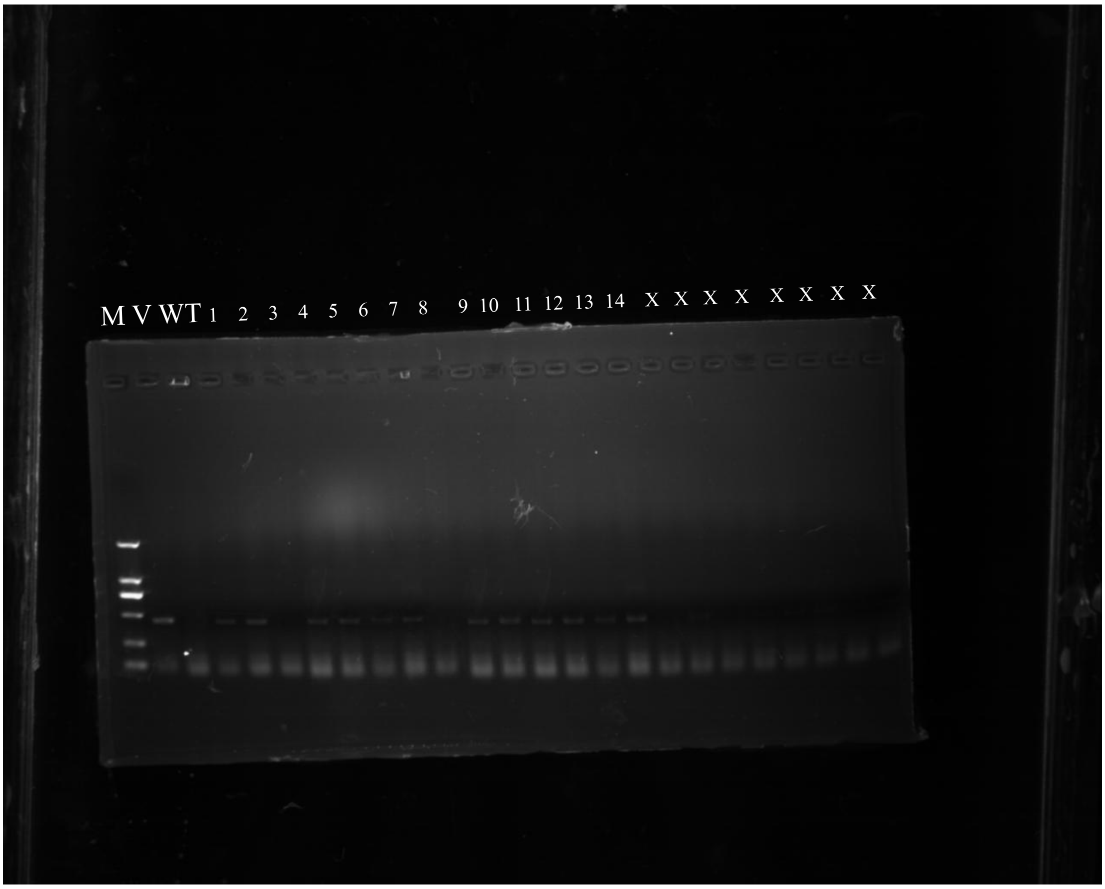

Fig 4. (B) Gel images of PCR products obtained with a set of primer pairs for the Cas9 of sgRNA/Cas9 vectors. M, DL2000 DNA marker. V, plasmid of CRISPR/Cas9 vector used in transformation. WT, DNA of wild type soybean plant. Labels 1–14, individual mutant plants.

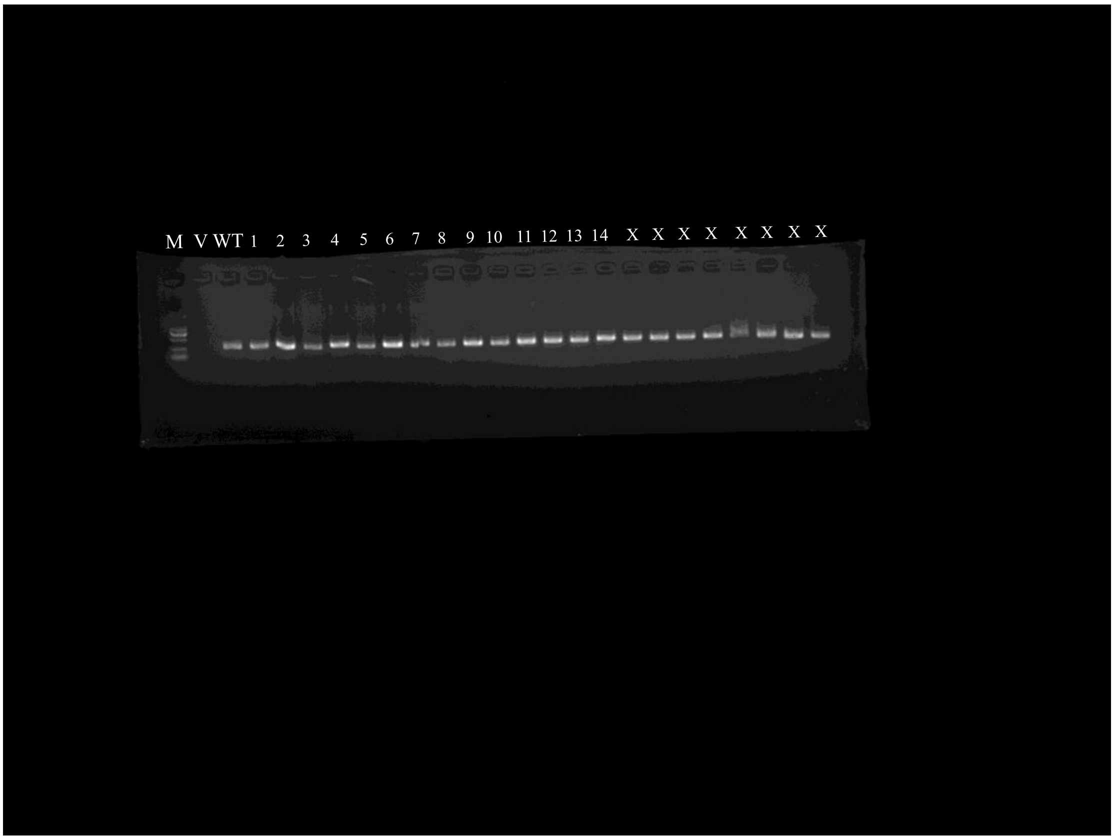

Fig 4. (B) Gel images of PCR products obtained with a set of primer pairs for the *GmActin* of soybean. M, DL2000 DNA marker. V, plasmid of CRISPR/Cas9 vector used in transformation. WT, DNA of wild type soybean plant. Labels 1–14, individual mutant plants.

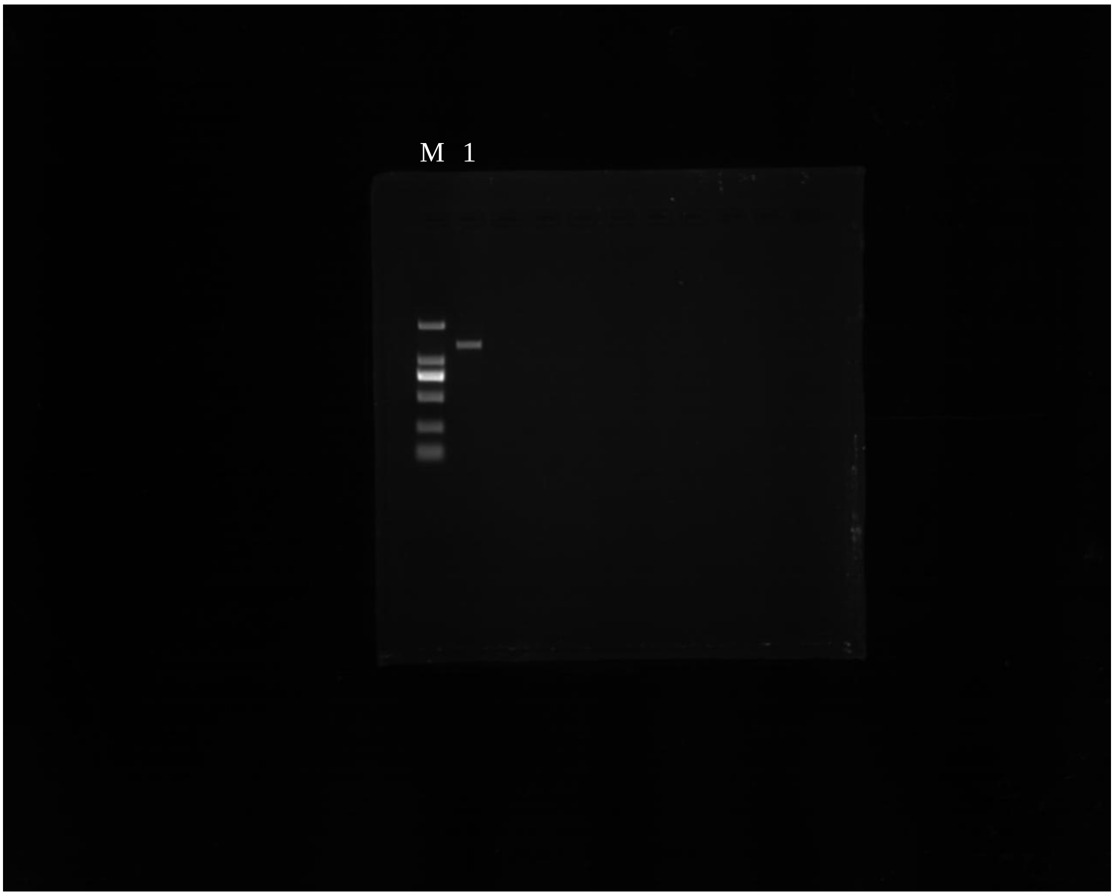

Fig S3. (A) The PCR amplified products of *GmTCP19L*. M, DL2000 DNA Marker. 1, *GmTCP19L*.
