# Supplementary figures and images for: CRISPR/Cas9-mediated targeted mutagenesis of GmTCP19L increasing susceptibility to Phytophthora sojae in soybean
Source: PLoS One. 2022 Jun 9;17(6):e0267502. doi: 10.1371/journal.pone.0267502 (PMC9182224; doi:10.1371/journal.pone.0267502)

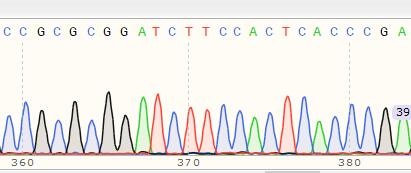

Supplement: S1 Data — (ZIP) [file pone.0267502.s010.zip › Figure 2B-GmTCP19L-SP1(1-bp deletion).jpg]

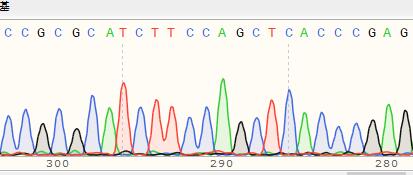

Supplement: S1 Data — (ZIP) [file pone.0267502.s010.zip › Figure 2B-GmTCP19L-SP1(2-bp deletion).jpg]

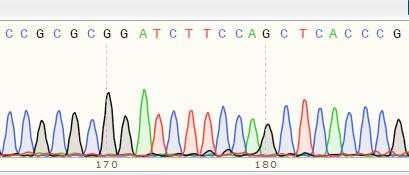

Supplement: S1 Data — (ZIP) [file pone.0267502.s010.zip › Figure 2B-GmTCP19L-SP1(WT).jpg]

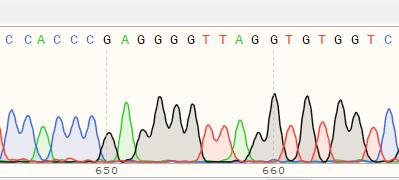

Supplement: S1 Data — (ZIP) [file pone.0267502.s010.zip › Figure 2C-GmTCP19L-SP2(14-bp deletion).jpg]

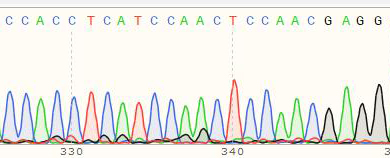

Supplement: S1 Data — (ZIP) [file pone.0267502.s010.zip › Figure 2C-GmTCP19L-SP2(WT).jpg]

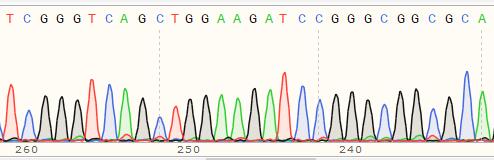

Supplement: S1 Data — (ZIP) [file pone.0267502.s010.zip › Figure 3-OFF1-WT.jpg]

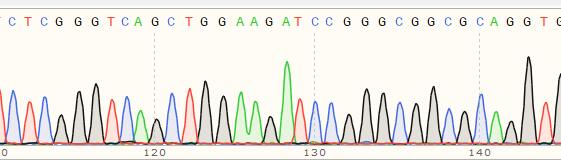

Supplement: S1 Data — (ZIP) [file pone.0267502.s010.zip › Figure 3-OFF1.jpg]

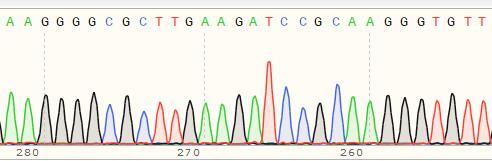

Supplement: S1 Data — (ZIP) [file pone.0267502.s010.zip › Figure 3-OFF2-WT.jpg]

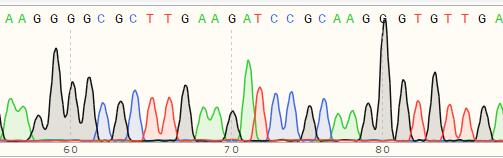

Supplement: S1 Data — (ZIP) [file pone.0267502.s010.zip › Figure 3-OFF2.jpg]

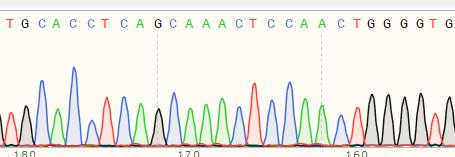

Supplement: S1 Data — (ZIP) [file pone.0267502.s010.zip › Figure 3-OFF3-WT.jpg]

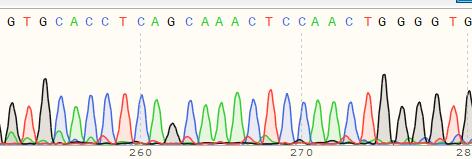

Supplement: S1 Data — (ZIP) [file pone.0267502.s010.zip › Figure 3-OFF3.jpg]

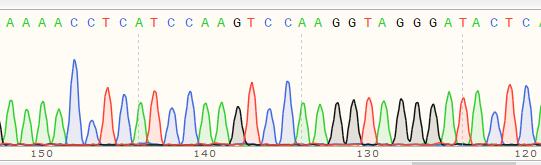

Supplement: S1 Data — (ZIP) [file pone.0267502.s010.zip › Figure 3-OFF4-WT.jpg]

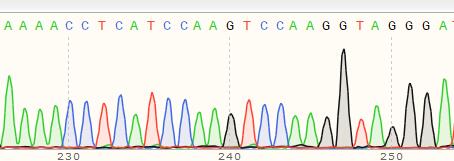

Supplement: S1 Data — (ZIP) [file pone.0267502.s010.zip › Figure 3-OFF4.jpg]

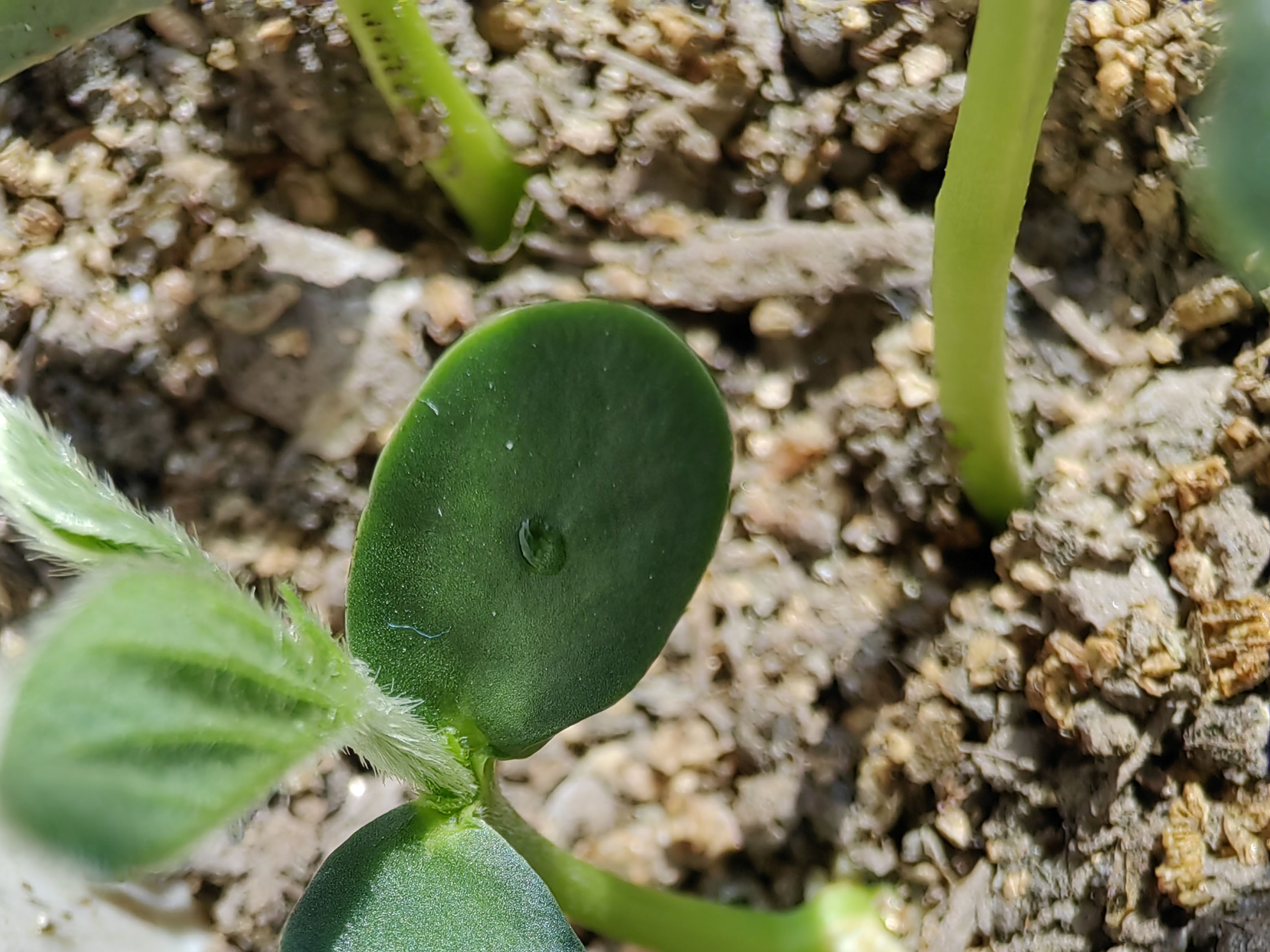

Supplement: S1 Data — (ZIP) [file pone.0267502.s010.zip › Figure 4A-0-1 bp deletion-1.jpg]

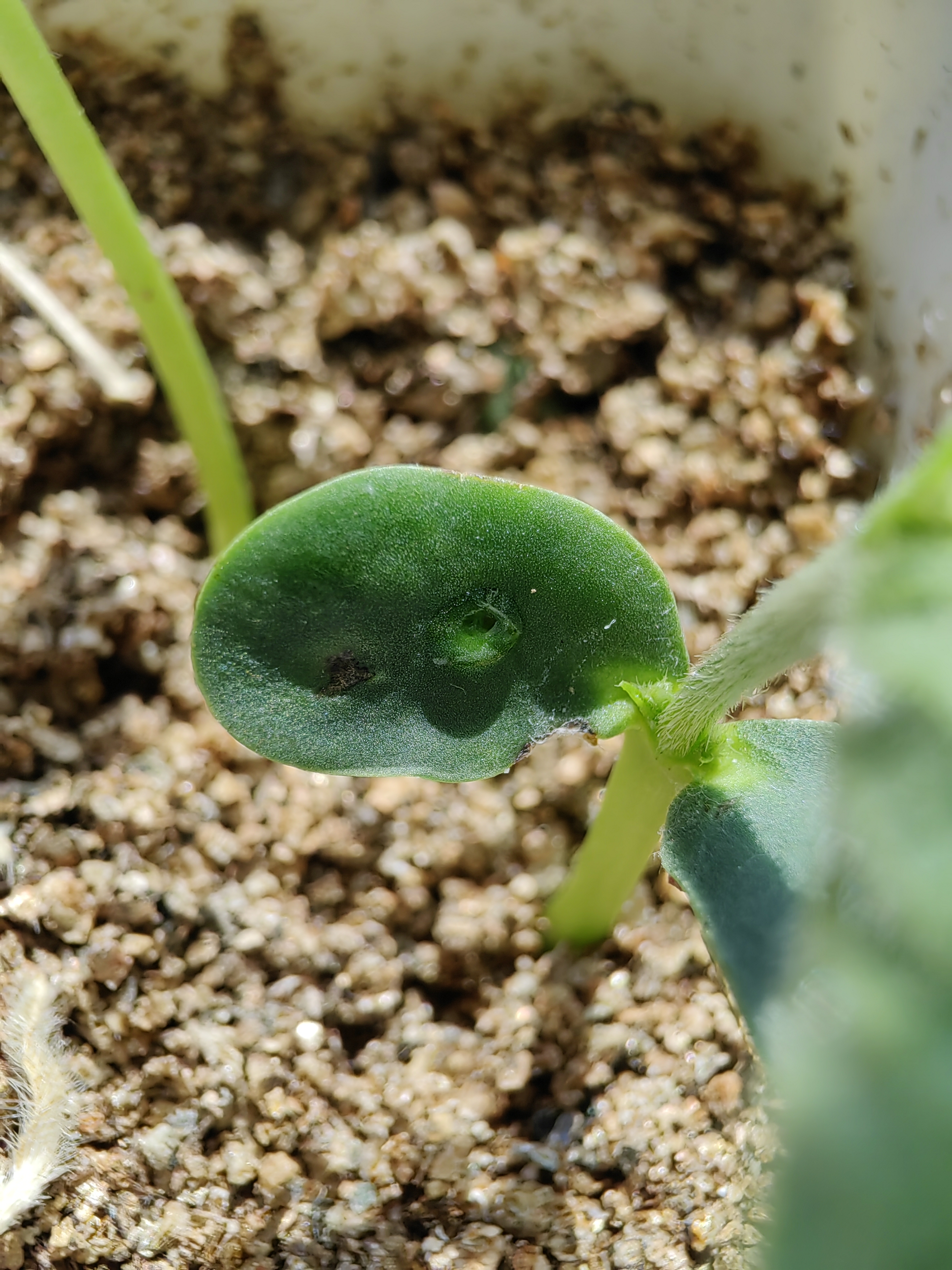

Supplement: S1 Data — (ZIP) [file pone.0267502.s010.zip › Figure 4A-0-1 bp deletion-2.jpg]

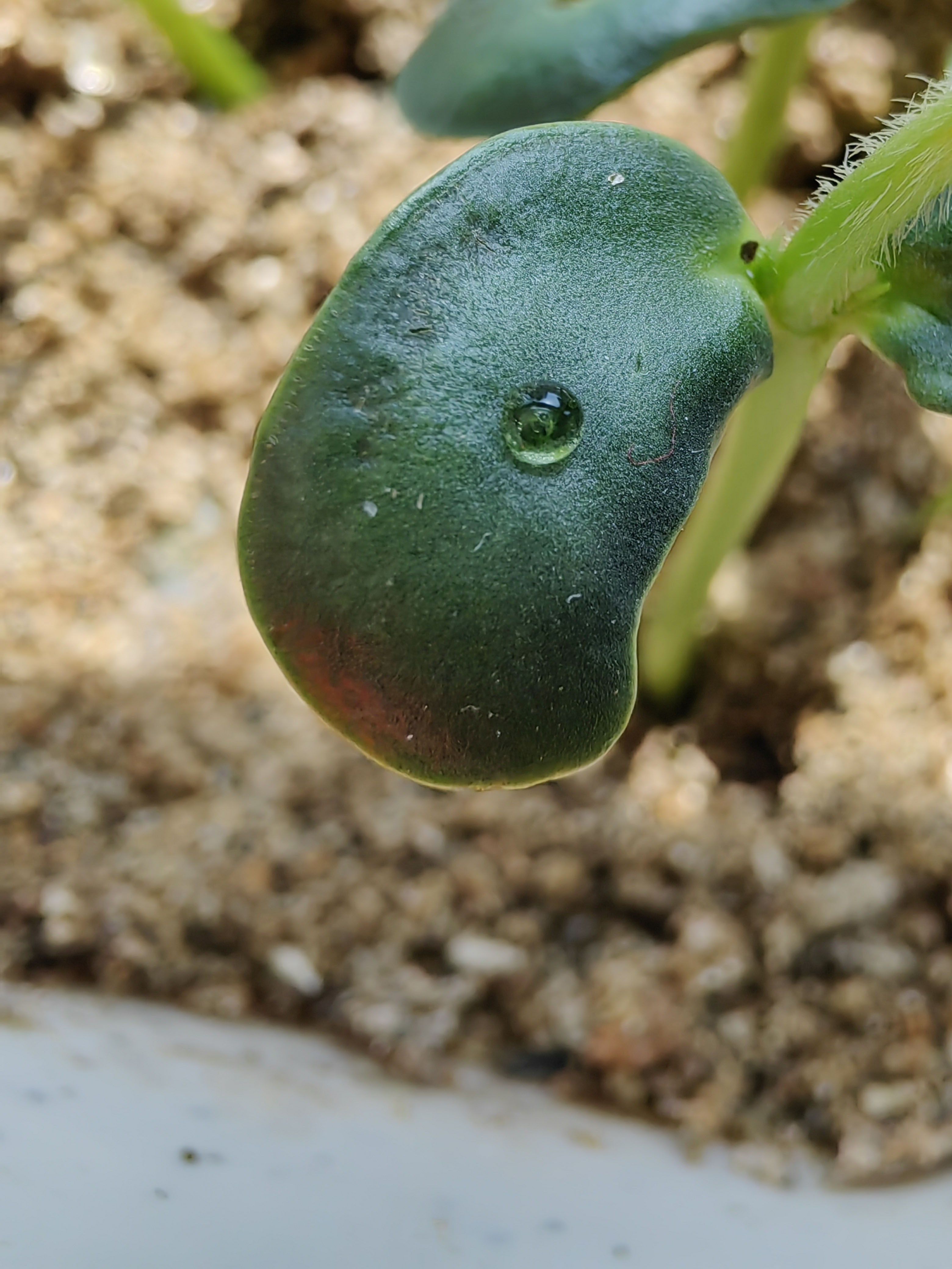

Supplement: S1 Data — (ZIP) [file pone.0267502.s010.zip › Figure 4A-0-14 bp deletion-1.jpg]

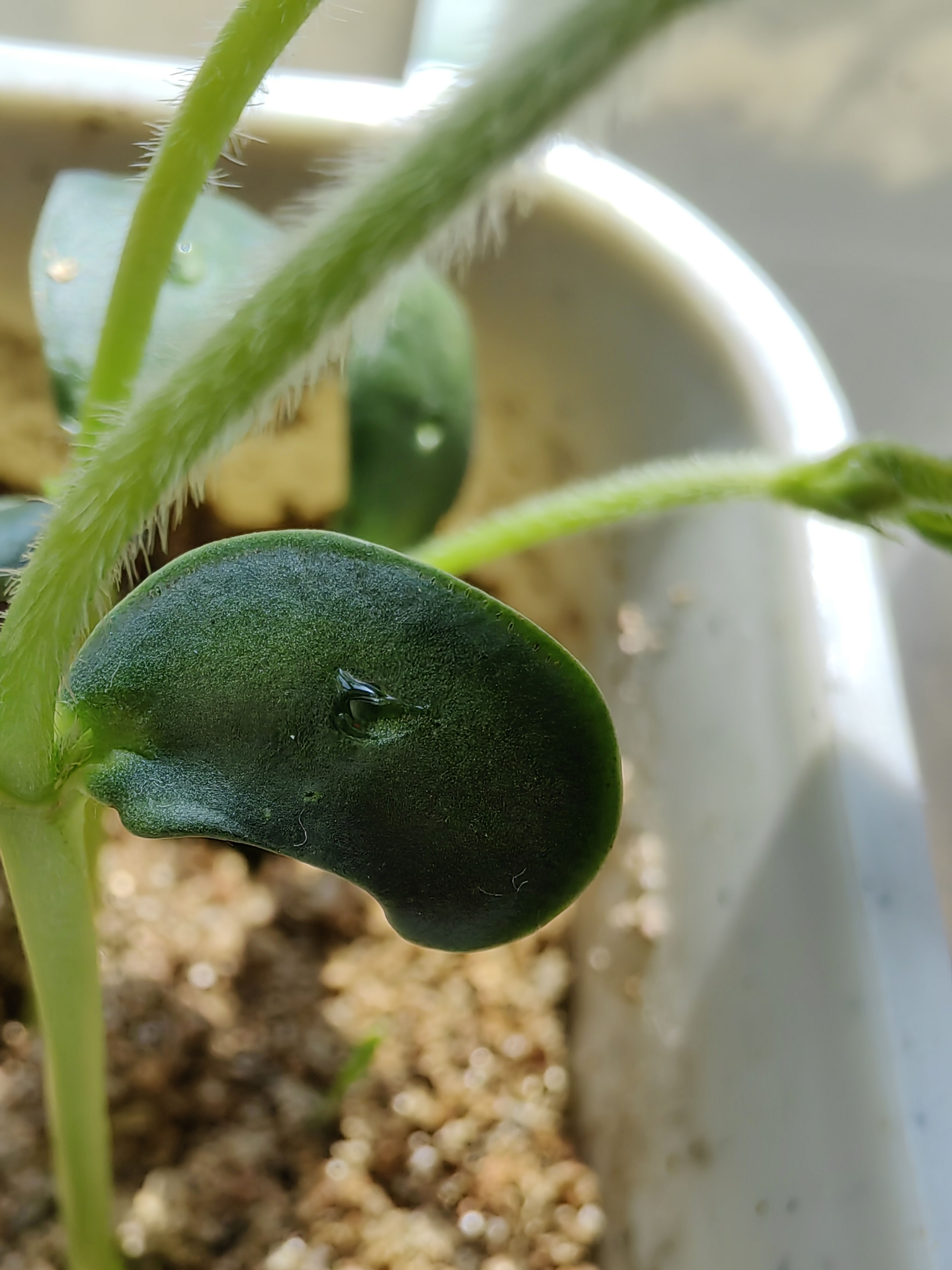

Supplement: S1 Data — (ZIP) [file pone.0267502.s010.zip › Figure 4A-0-14 bp deletion-2.jpg]

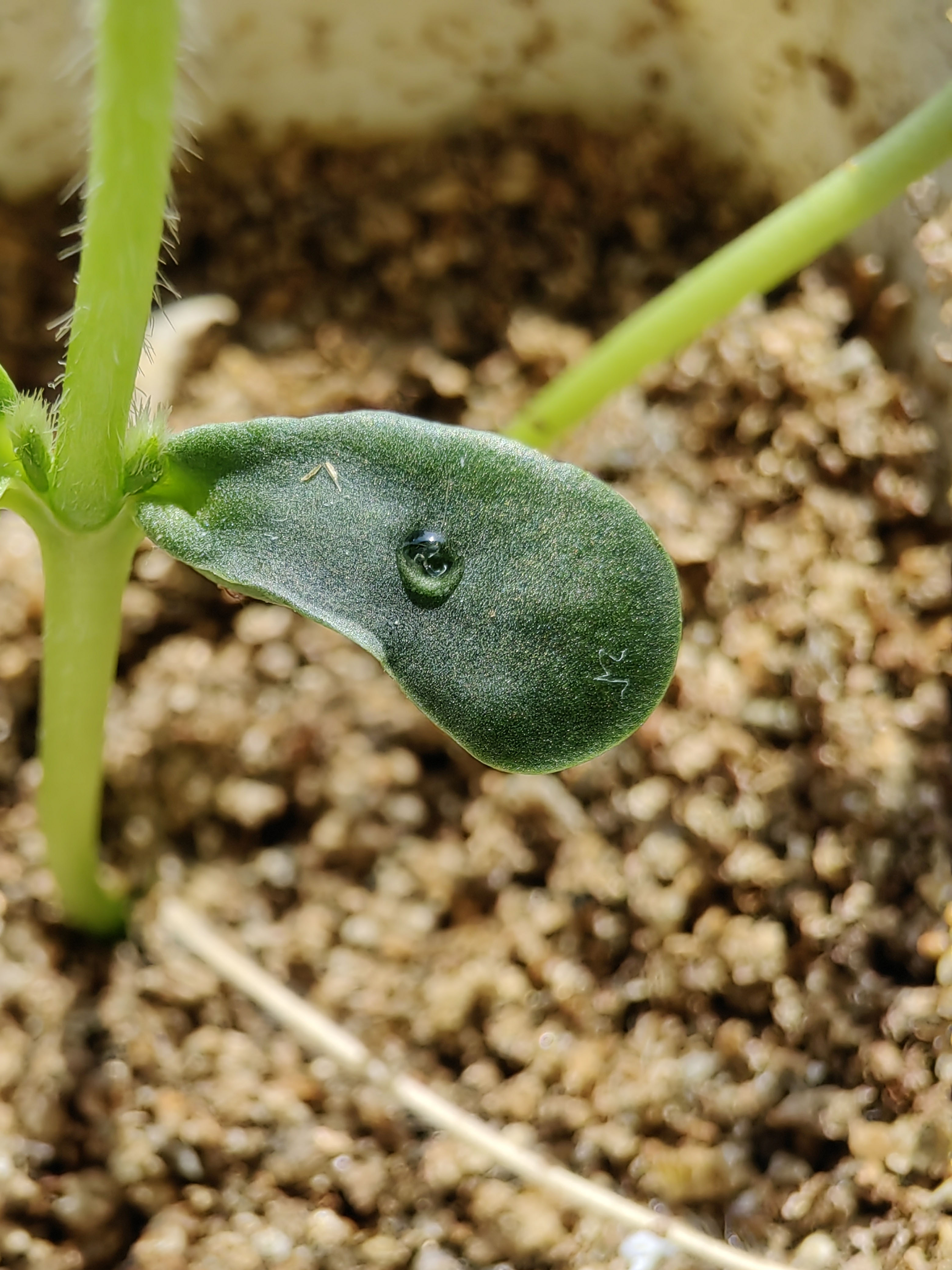

Supplement: S1 Data — (ZIP) [file pone.0267502.s010.zip › Figure 4A-0-2 bp deletion-1.jpg]

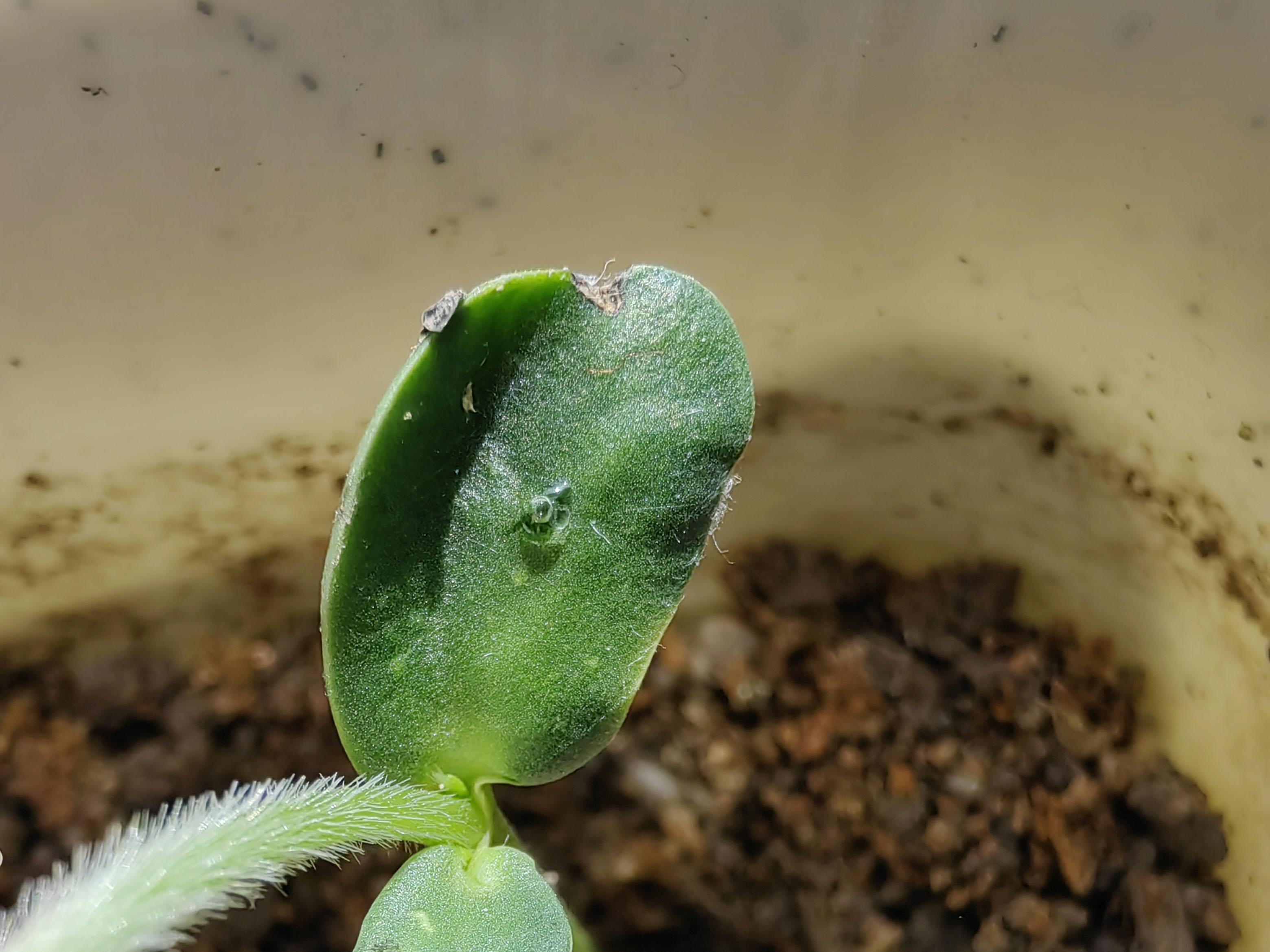

Supplement: S1 Data — (ZIP) [file pone.0267502.s010.zip › Figure 4A-0-2 bp deletion-2.jpg]

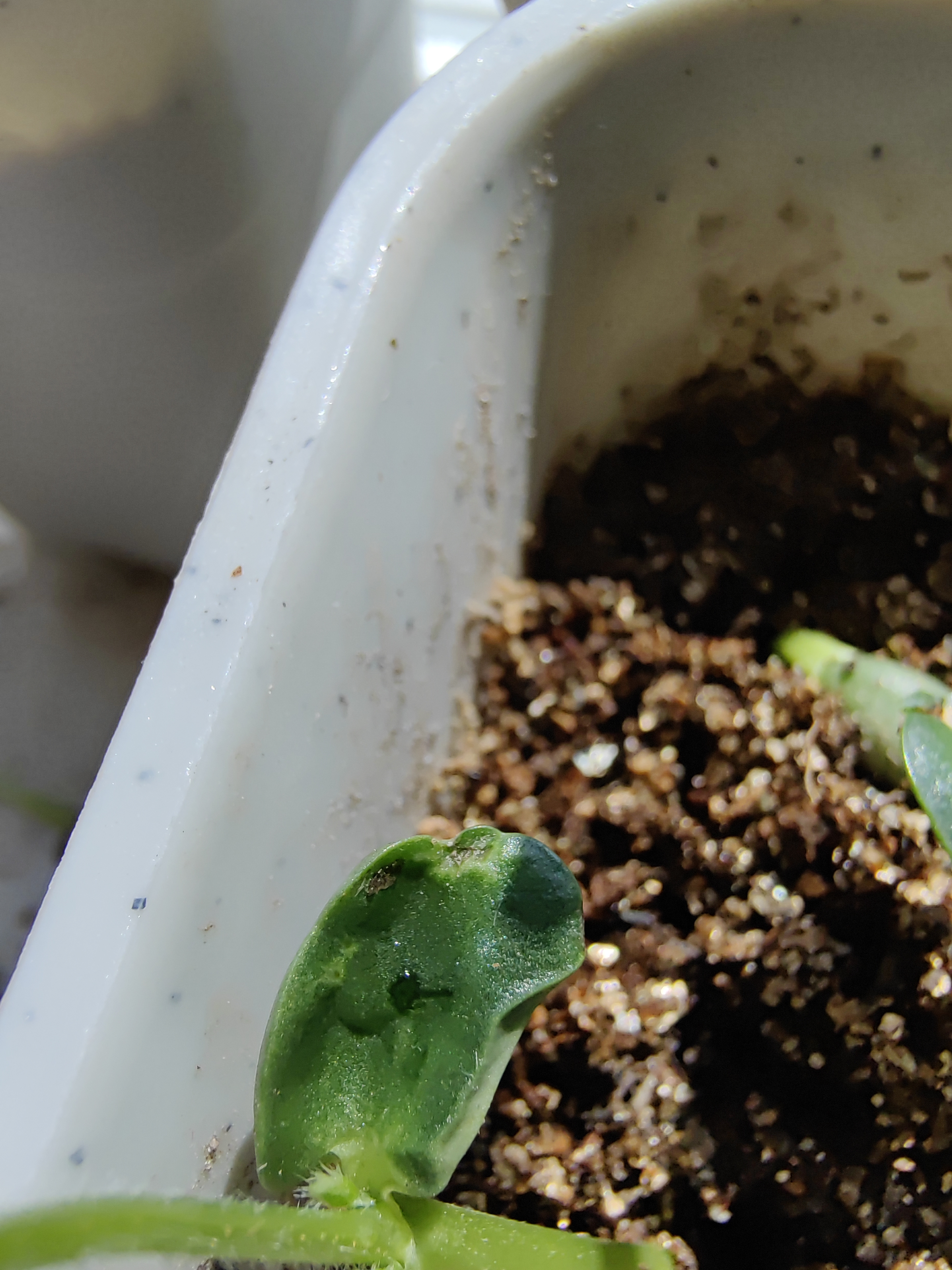

Supplement: S1 Data — (ZIP) [file pone.0267502.s010.zip › Figure 4A-0-WT-1.jpg]

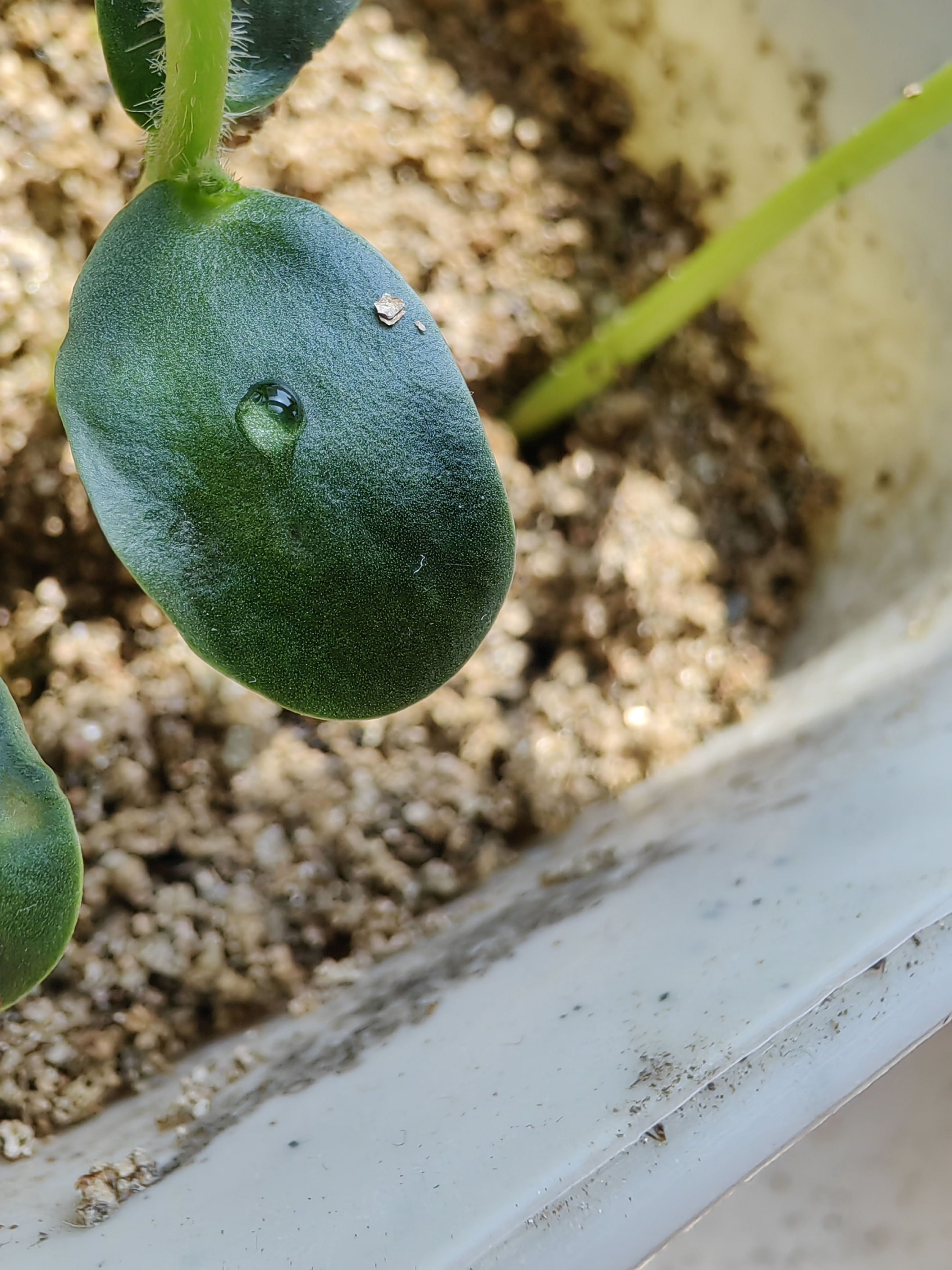

Supplement: S1 Data — (ZIP) [file pone.0267502.s010.zip › Figure 4A-0-WT-2.jpg]

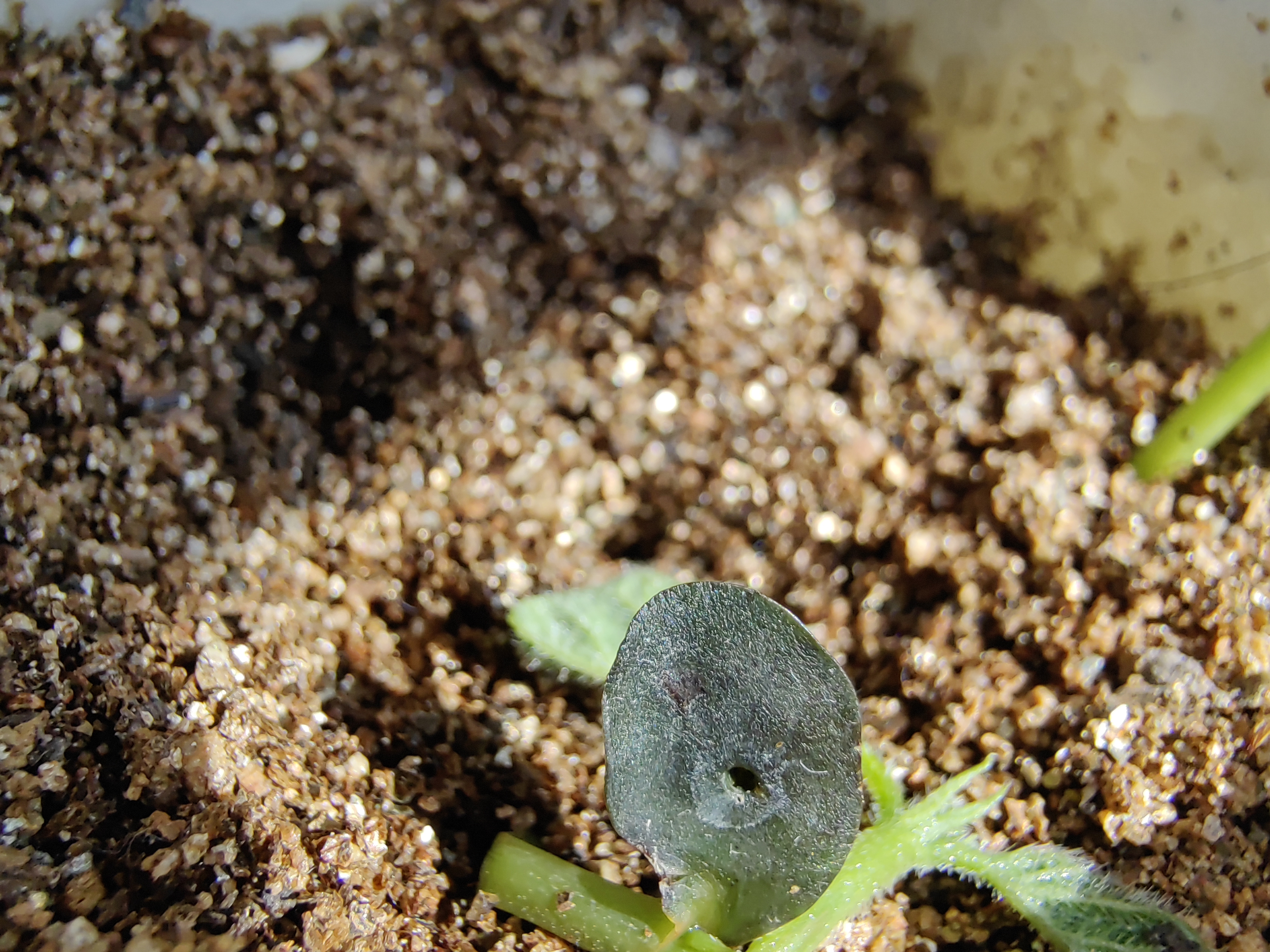

Supplement: S1 Data — (ZIP) [file pone.0267502.s010.zip › Figure 4A-3-1 bp deletion-1.jpg]

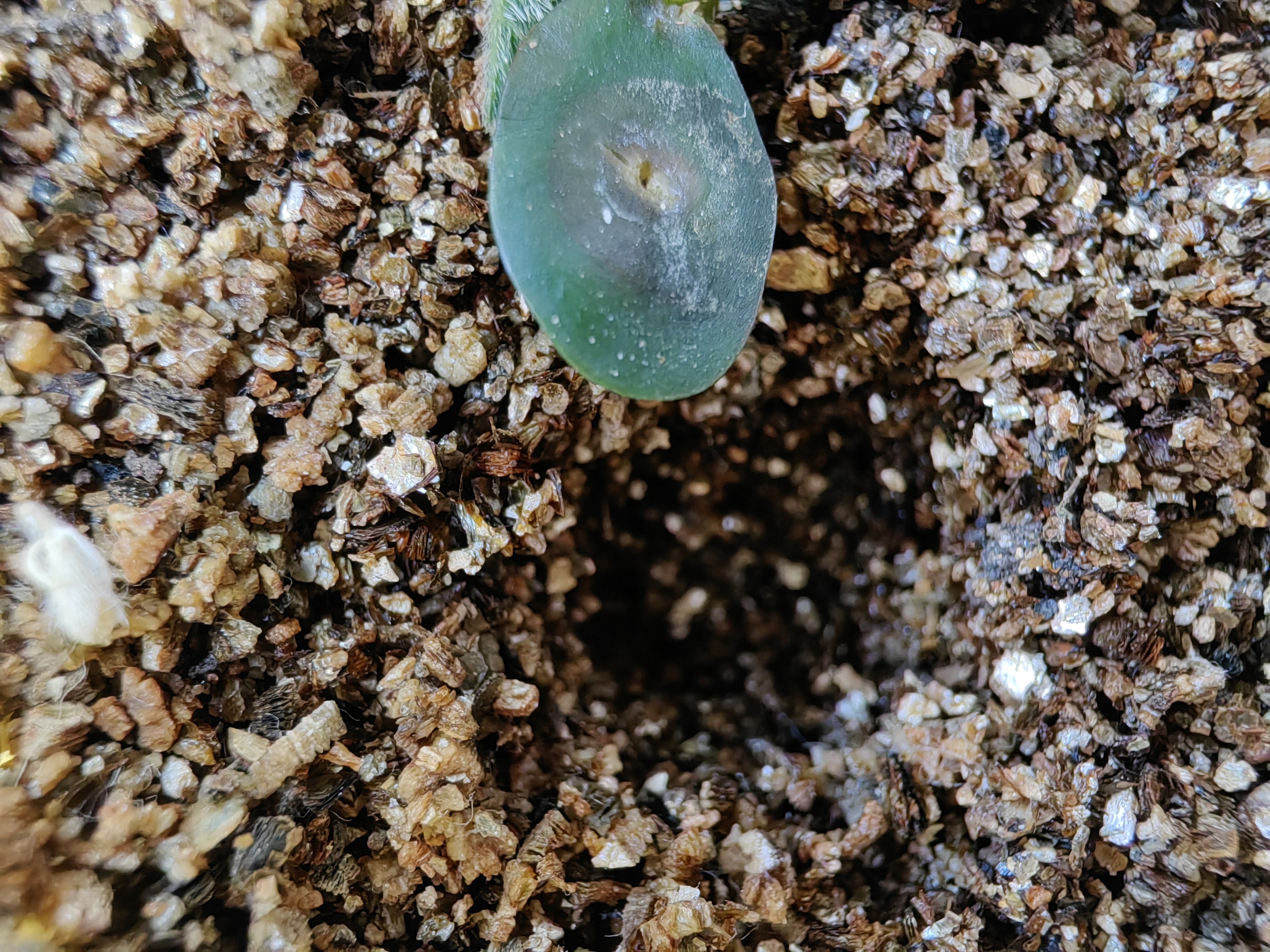

Supplement: S1 Data — (ZIP) [file pone.0267502.s010.zip › Figure 4A-3-1 bp deletion-2.jpg]

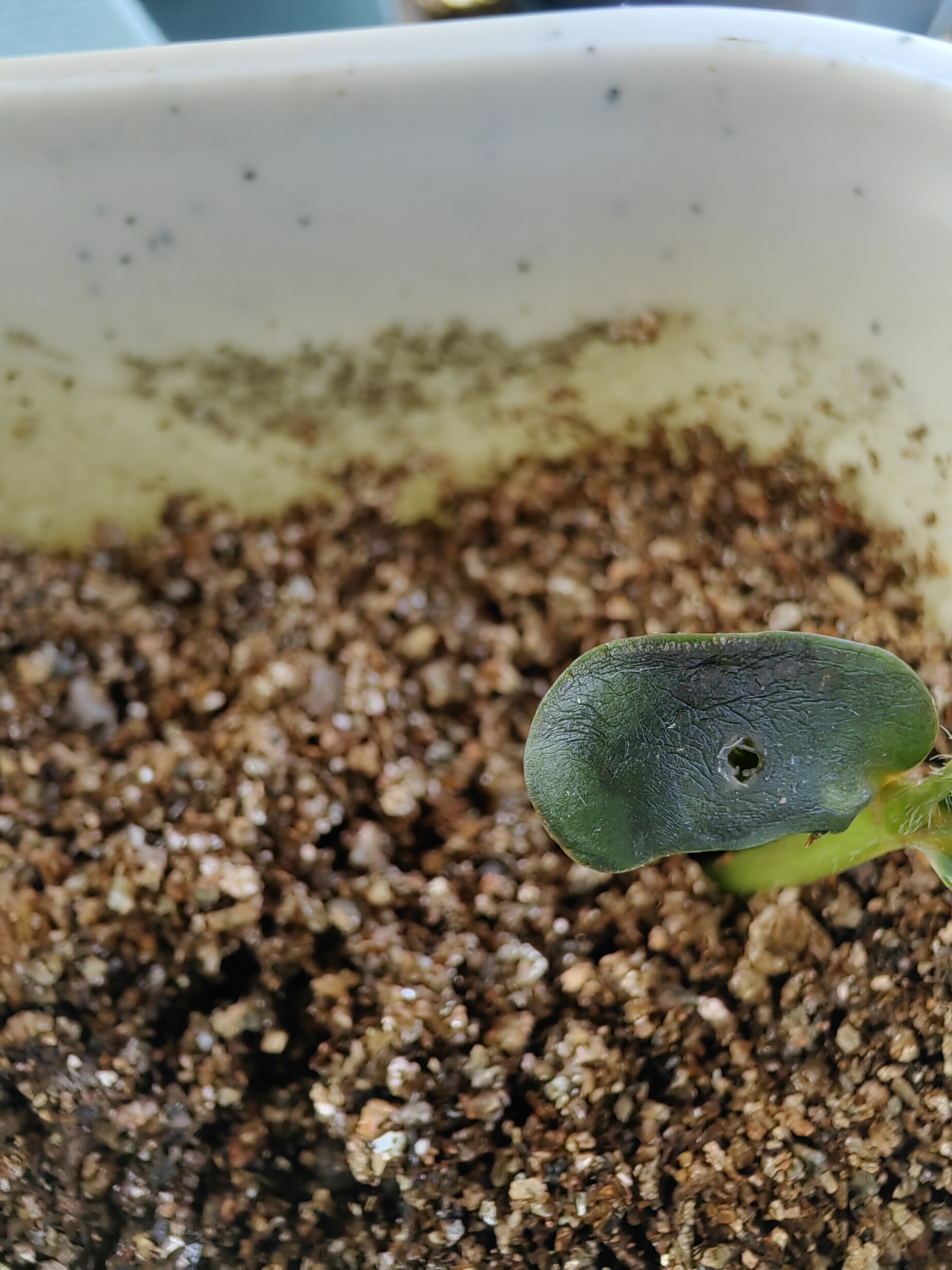

Supplement: S1 Data — (ZIP) [file pone.0267502.s010.zip › Figure 4A-3-14 bp deletion-1.jpg]

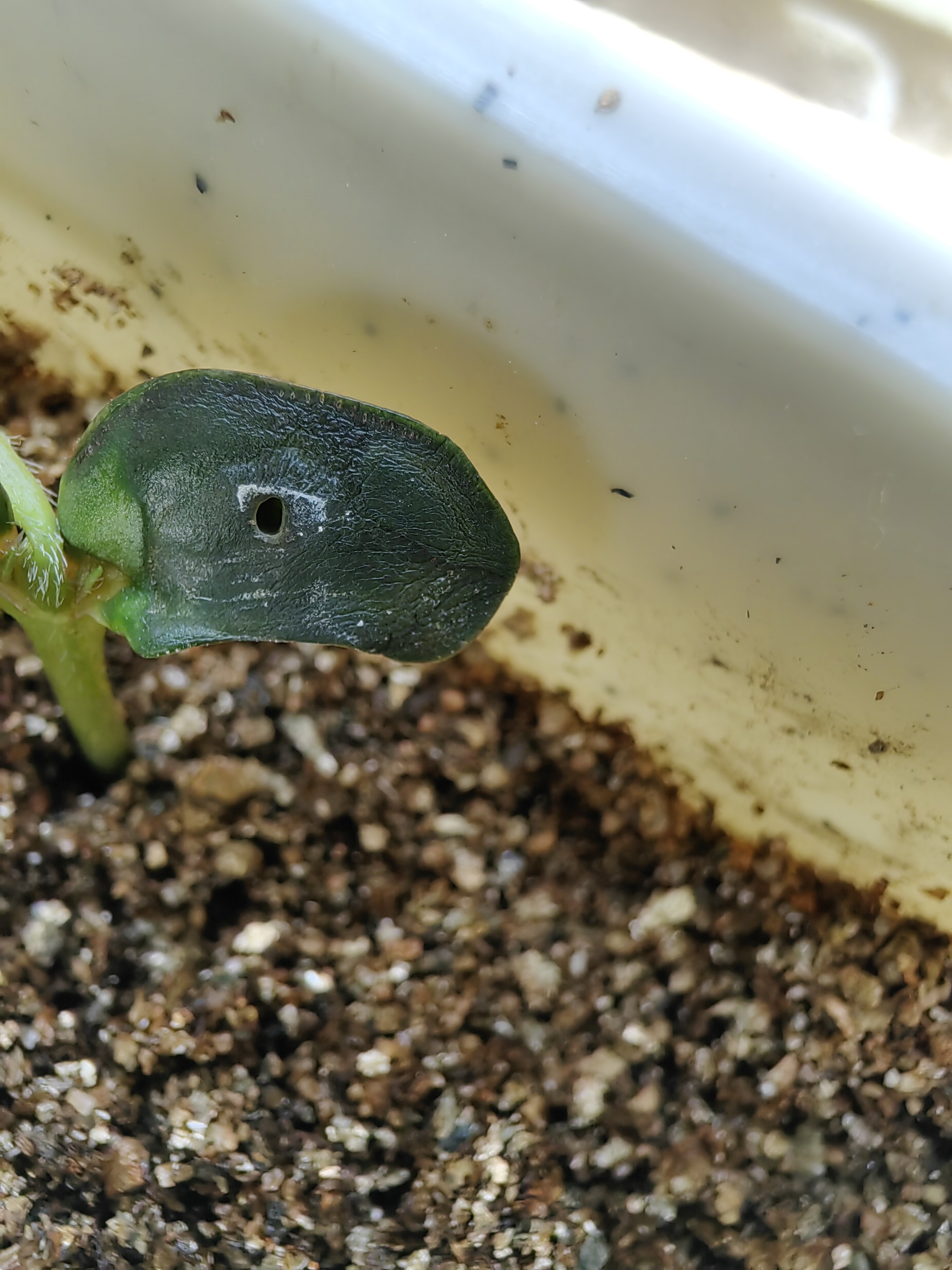

Supplement: S1 Data — (ZIP) [file pone.0267502.s010.zip › Figure 4A-3-14 bp deletion-2.jpg]

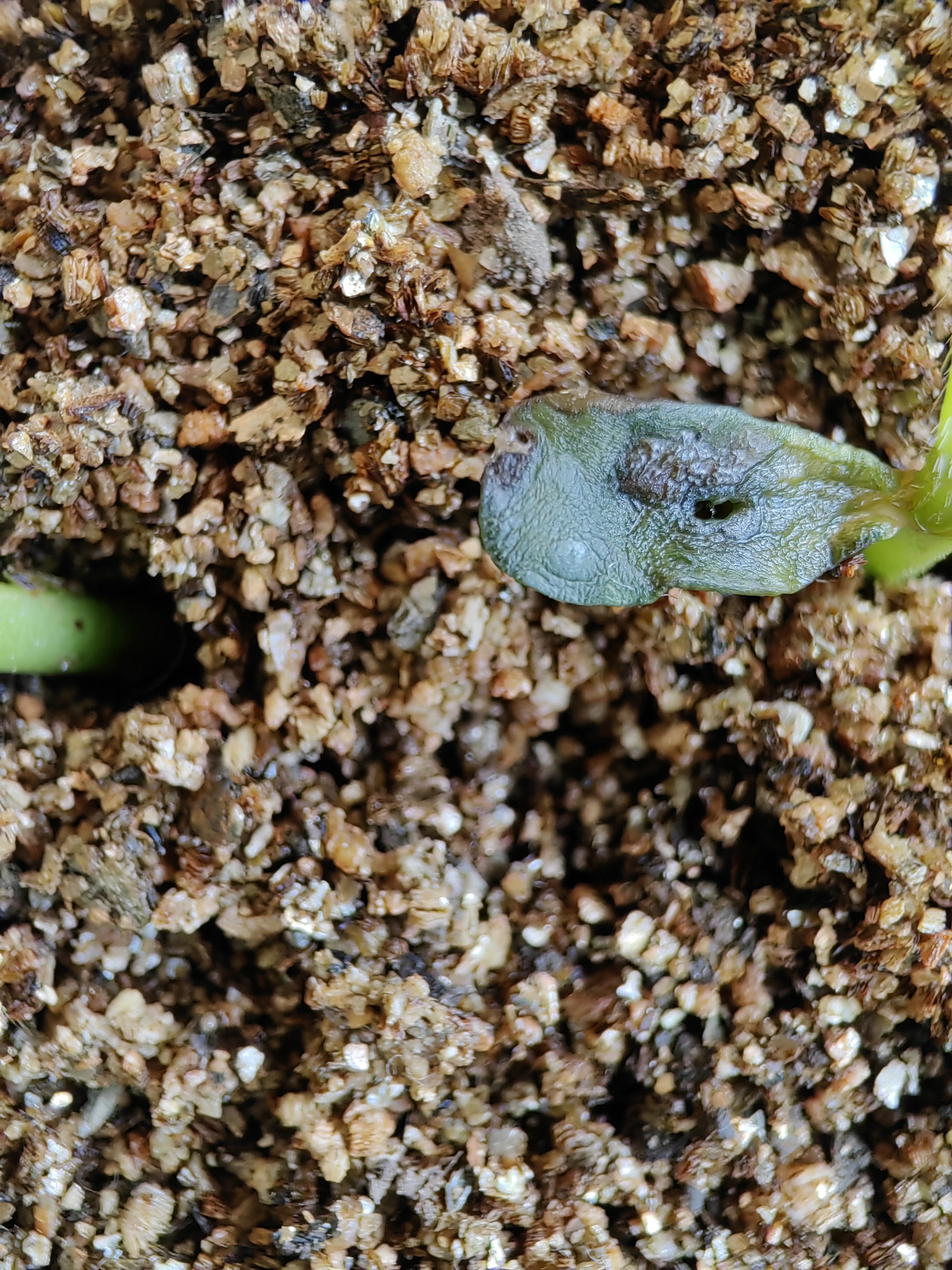

Supplement: S1 Data — (ZIP) [file pone.0267502.s010.zip › Figure 4A-3-2 bp deletion-1.jpg]

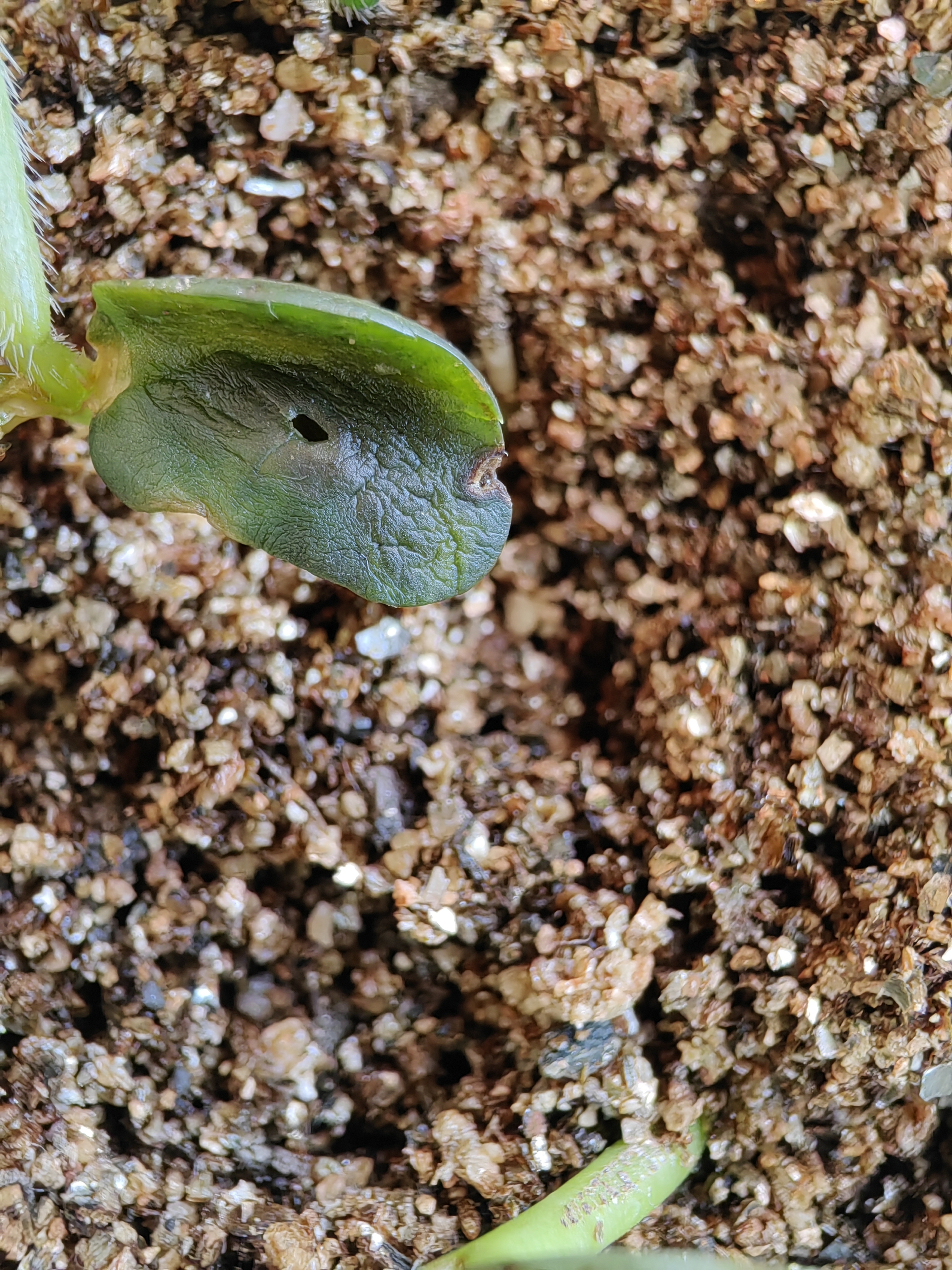

Supplement: S1 Data — (ZIP) [file pone.0267502.s010.zip › Figure 4A-3-2 bp deletion-2.jpg]

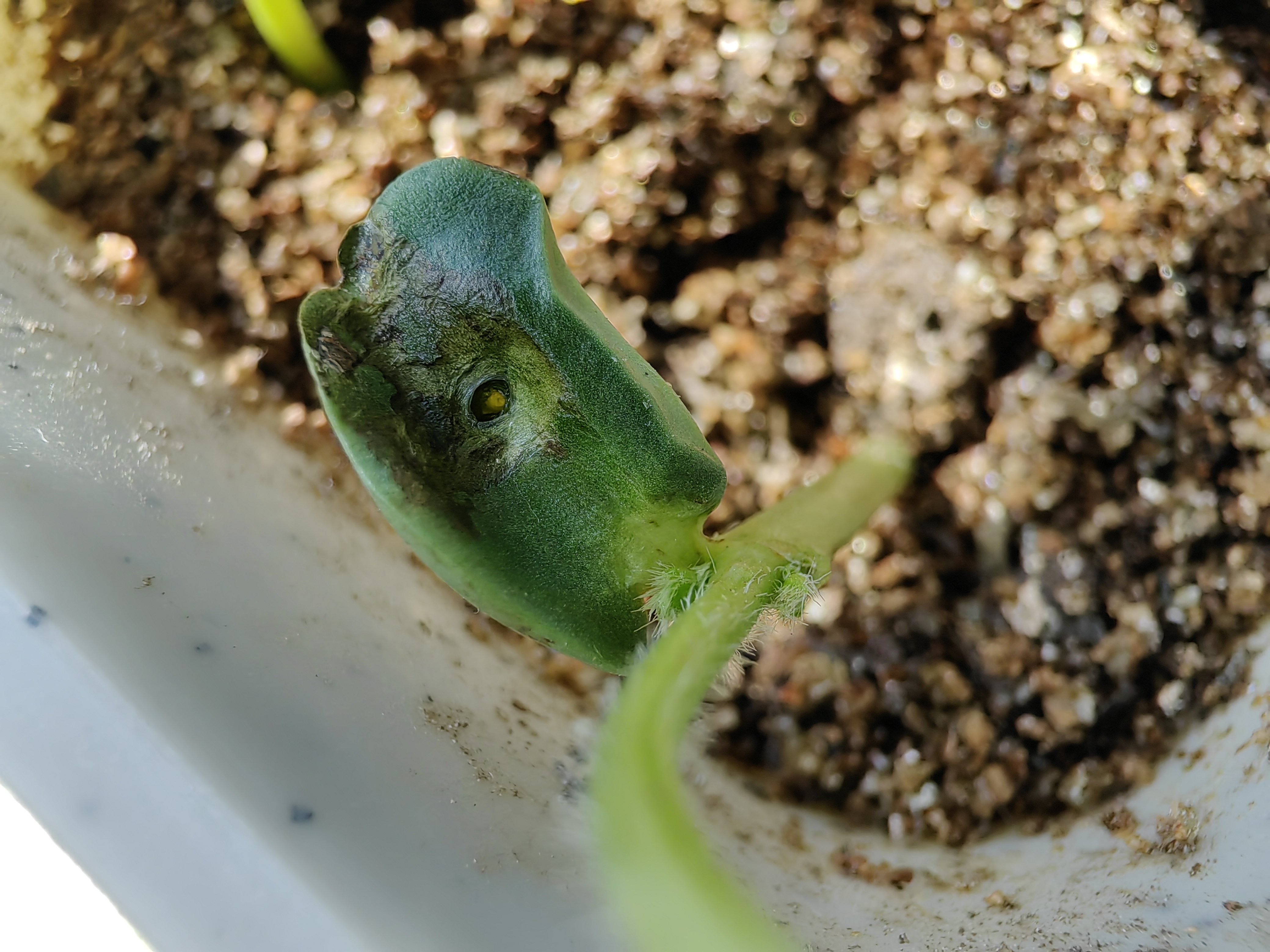

Supplement: S1 Data — (ZIP) [file pone.0267502.s010.zip › Figure 4A-3-WT-1.jpg]

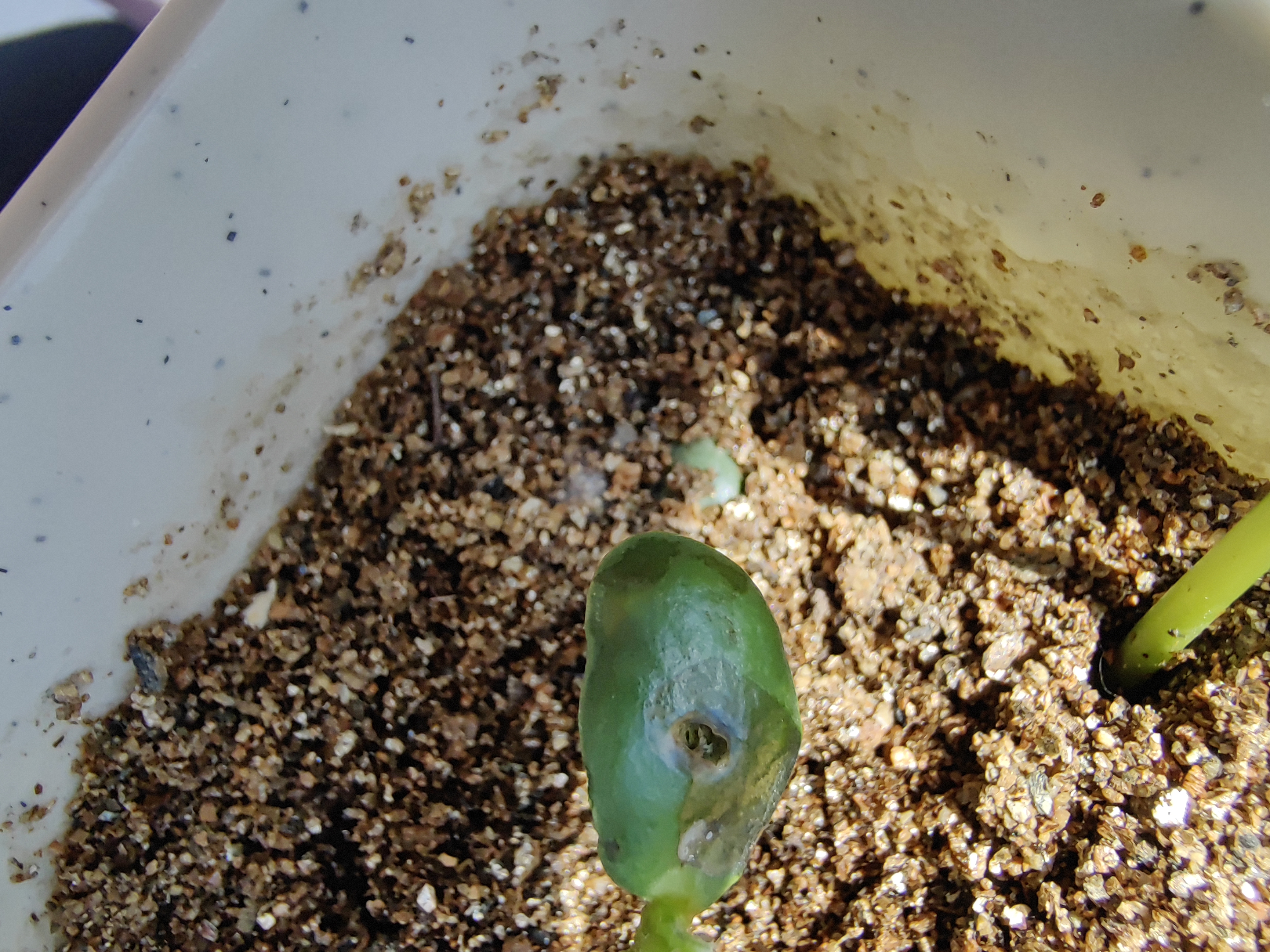

Supplement: S1 Data — (ZIP) [file pone.0267502.s010.zip › Figure 4A-3-WT-2.jpg]

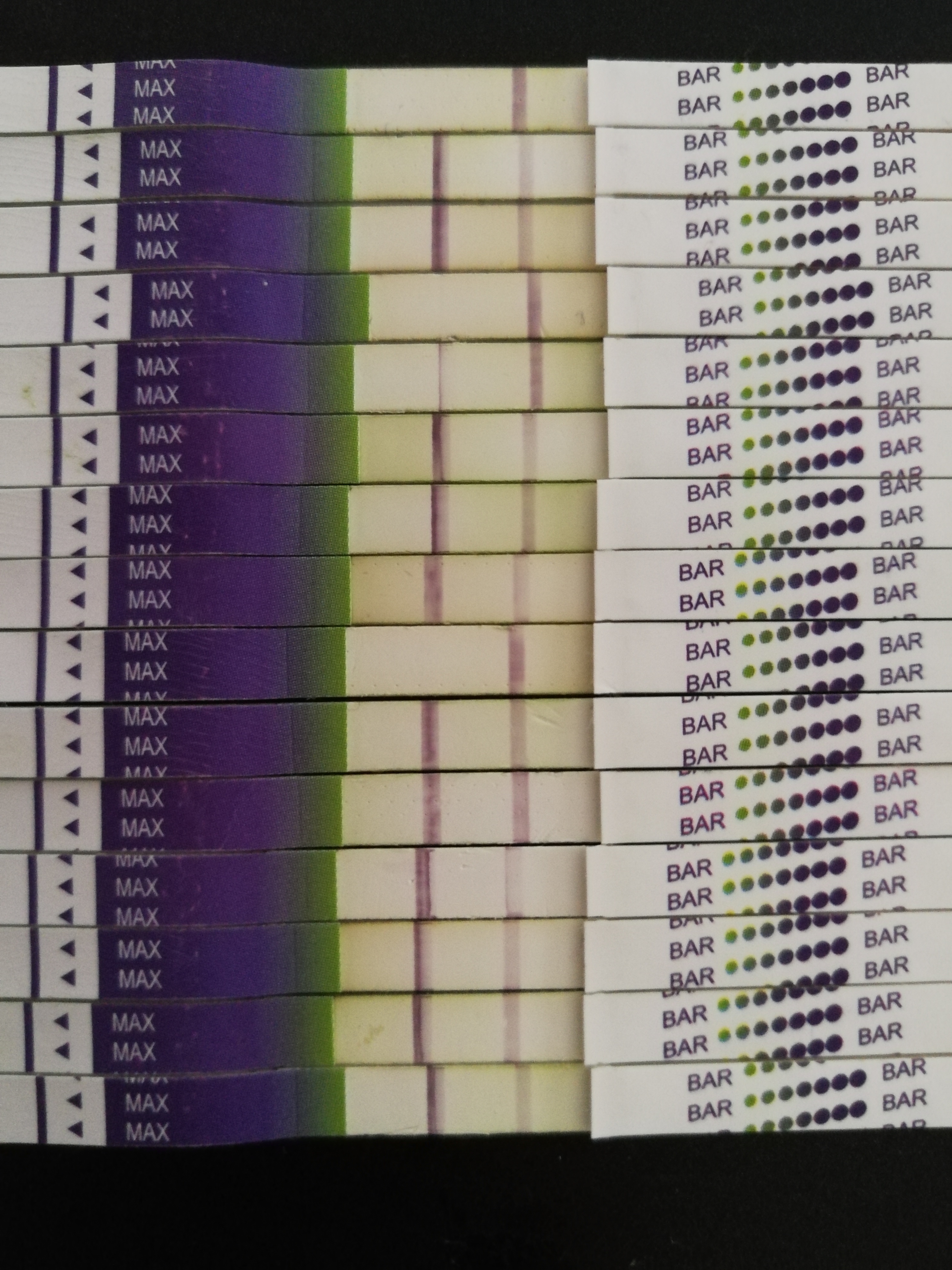

Supplement: S1 Data — (ZIP) [file pone.0267502.s010.zip › Figure 4A.jpg]

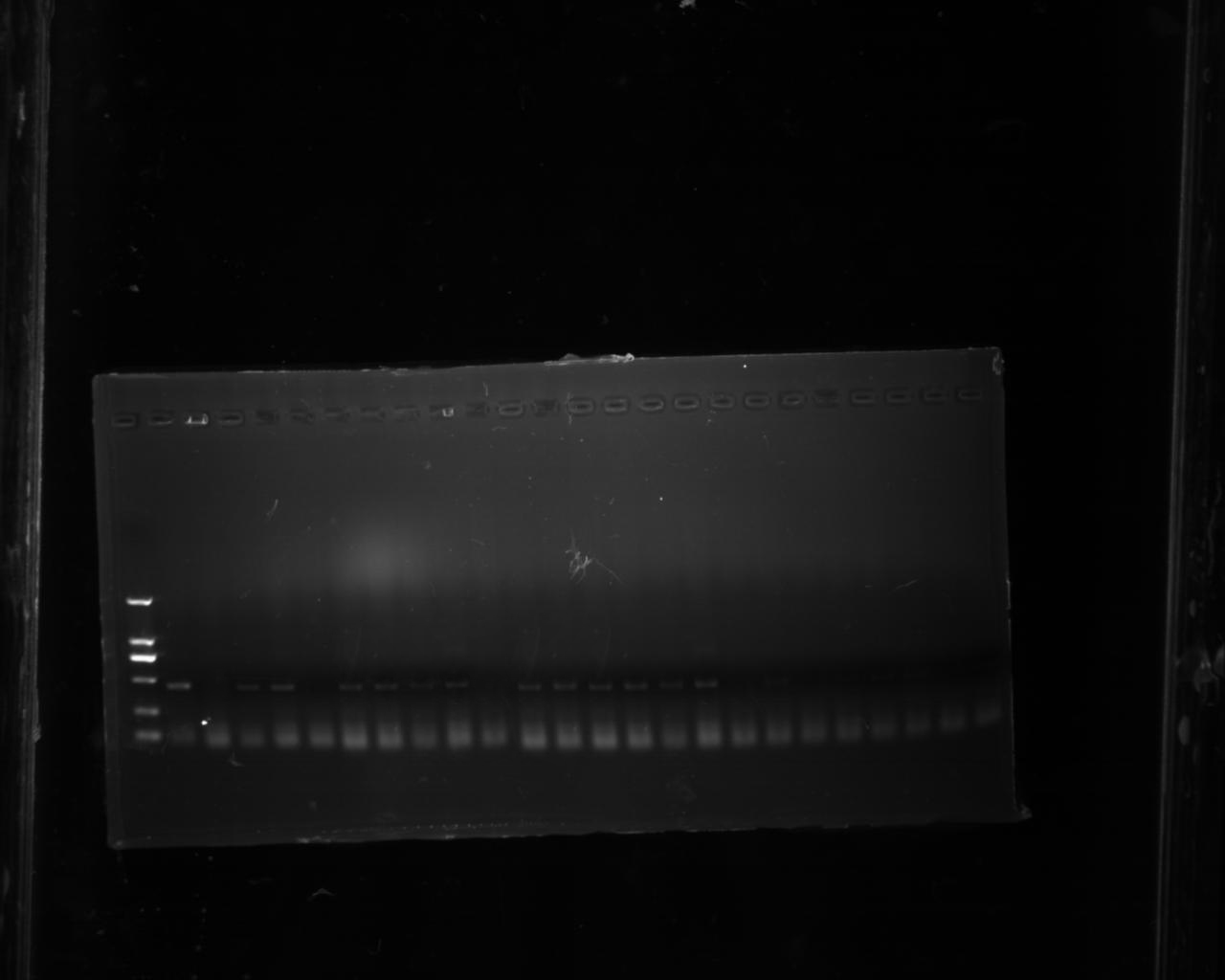

Supplement: S1 Data — (ZIP) [file pone.0267502.s010.zip › Figure 4B-Cas9.jpg]

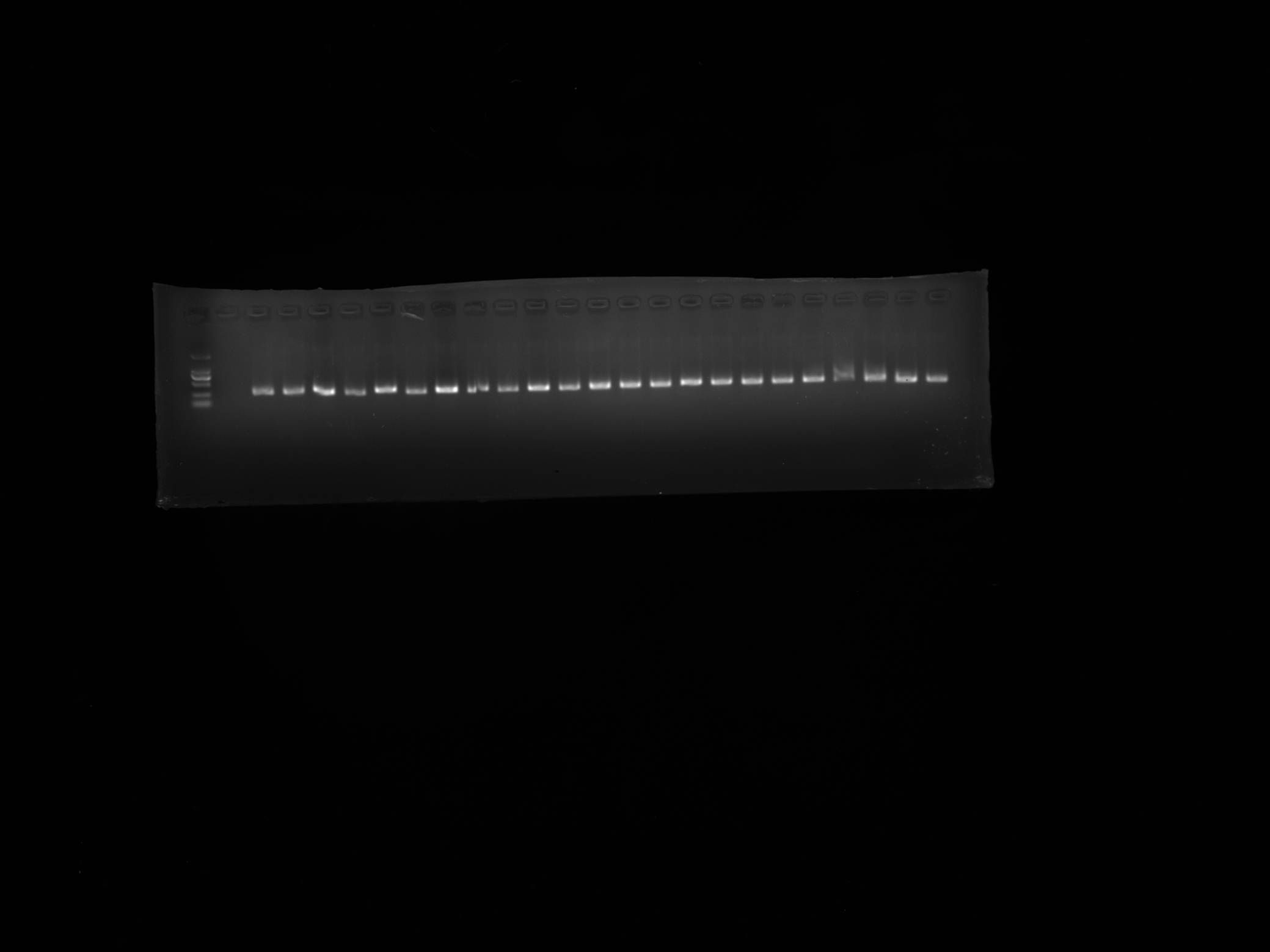

Supplement: S1 Data — (ZIP) [file pone.0267502.s010.zip › Figure 4B-GmActin.jpg]

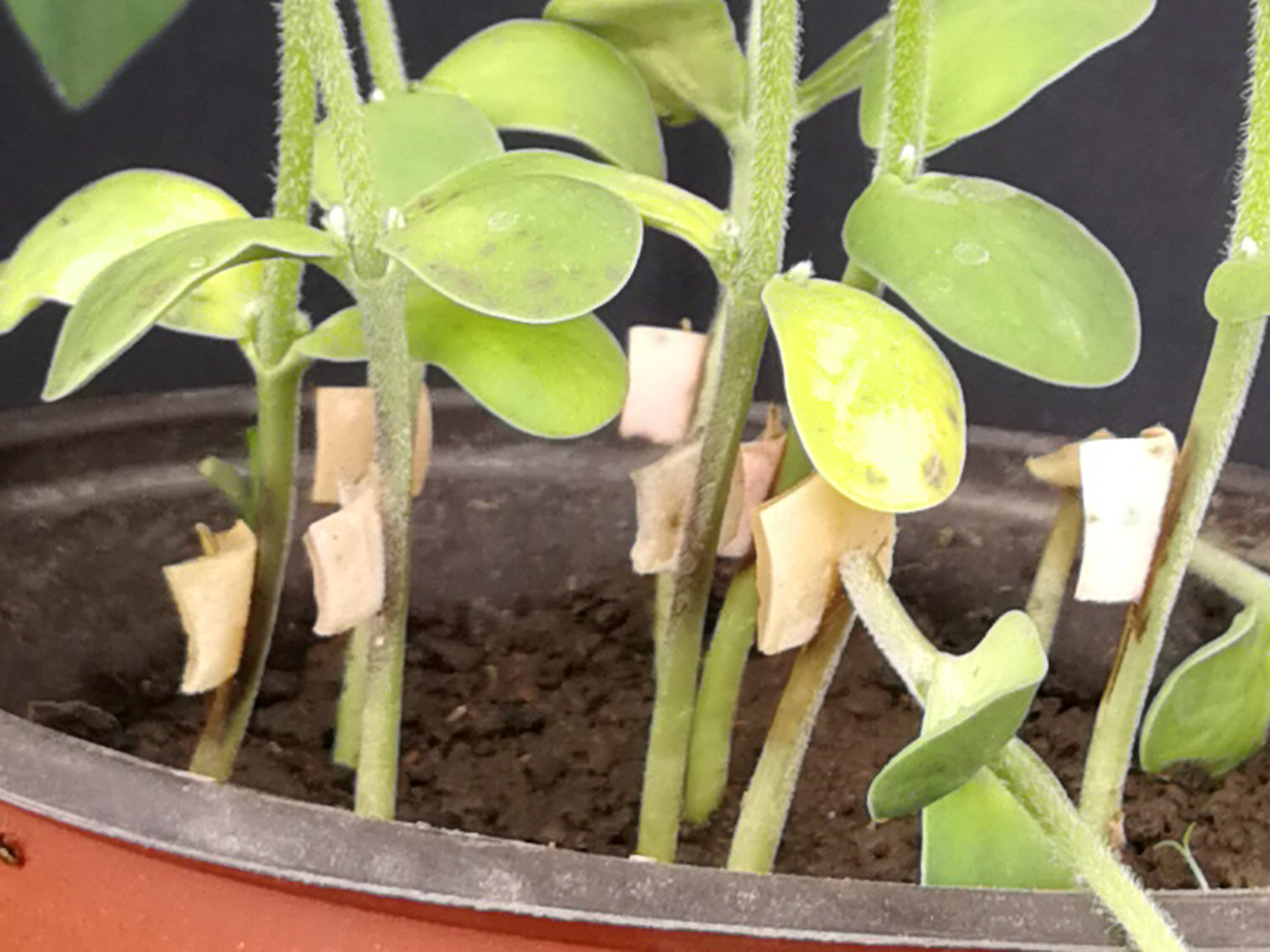

Supplement: S1 Data — (ZIP) [file pone.0267502.s010.zip › Figure 5-1.jpg]

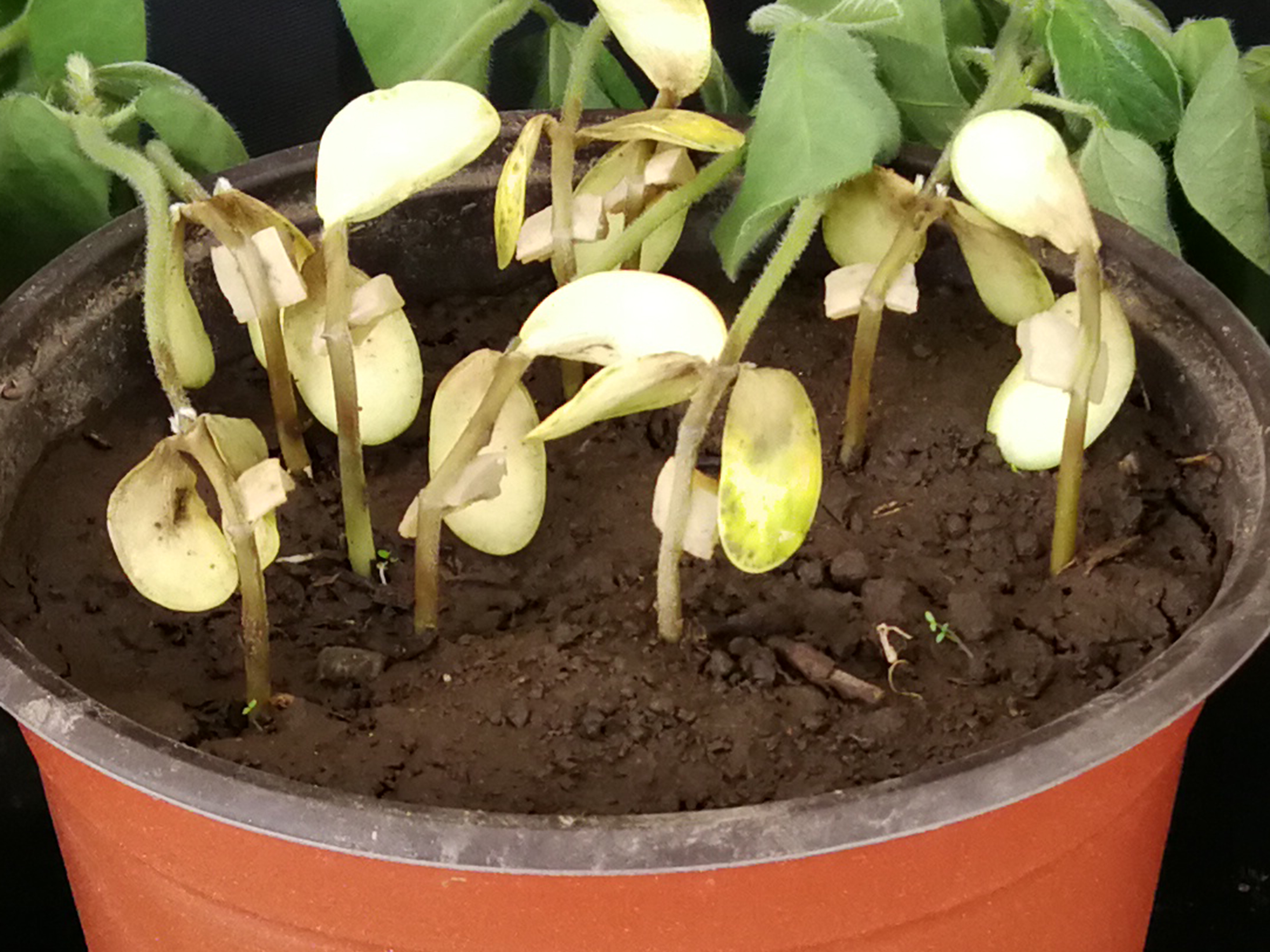

Supplement: S1 Data — (ZIP) [file pone.0267502.s010.zip › Figure 5-2.jpg]

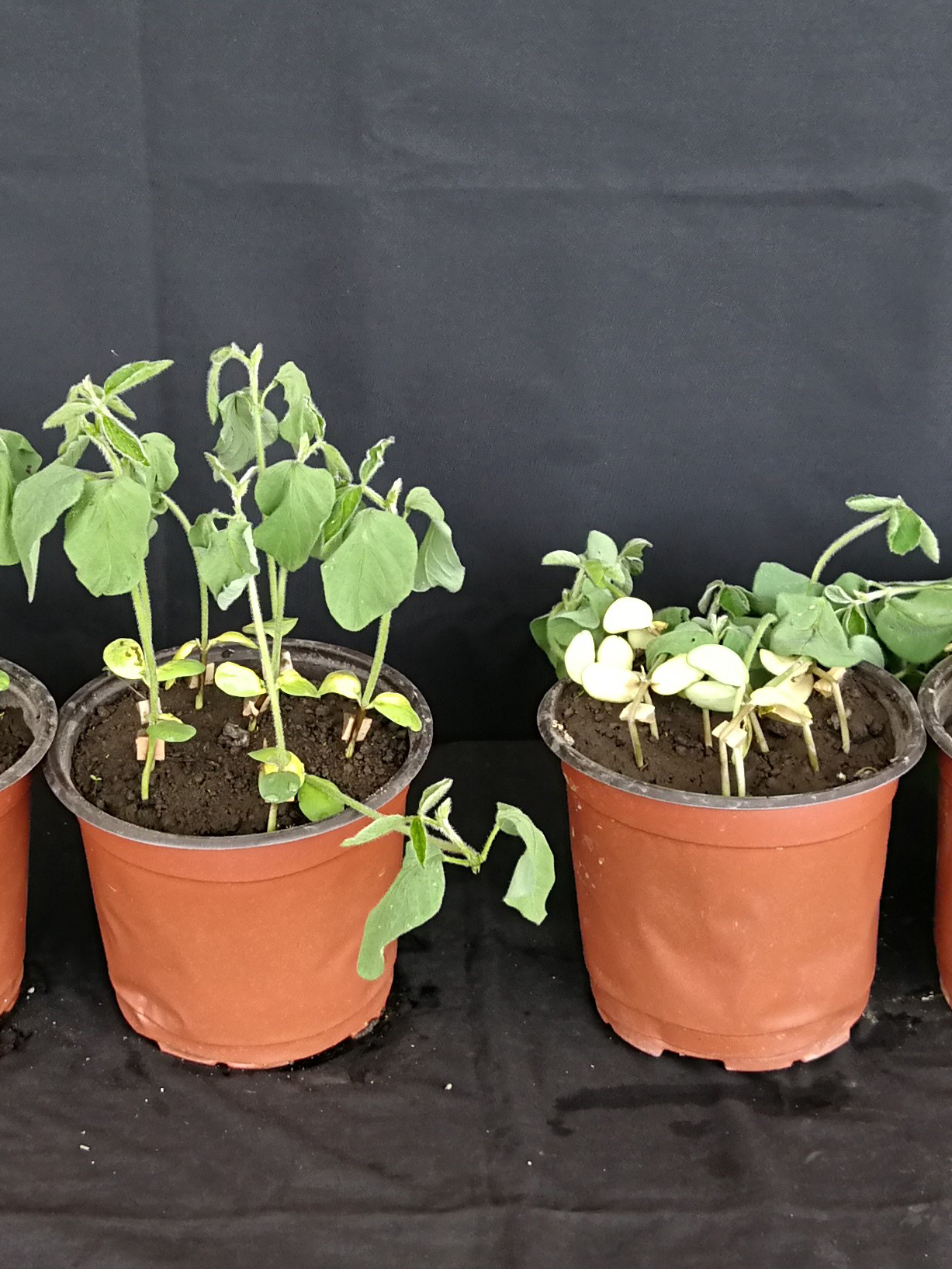

Supplement: S1 Data — (ZIP) [file pone.0267502.s010.zip › Figure 5-3.jpg]

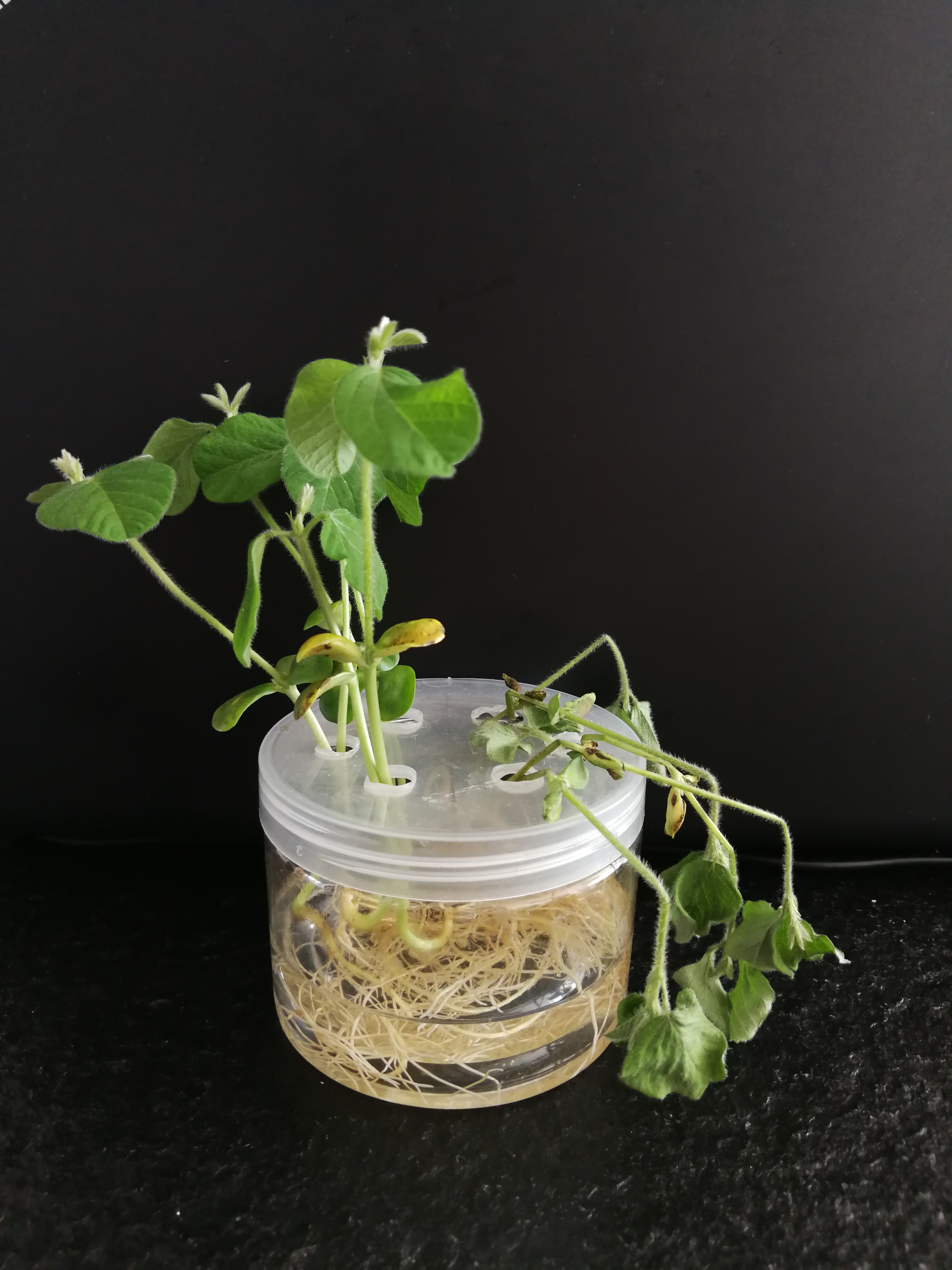

Supplement: S1 Data — (ZIP) [file pone.0267502.s010.zip › Figure 6A-1.jpg]

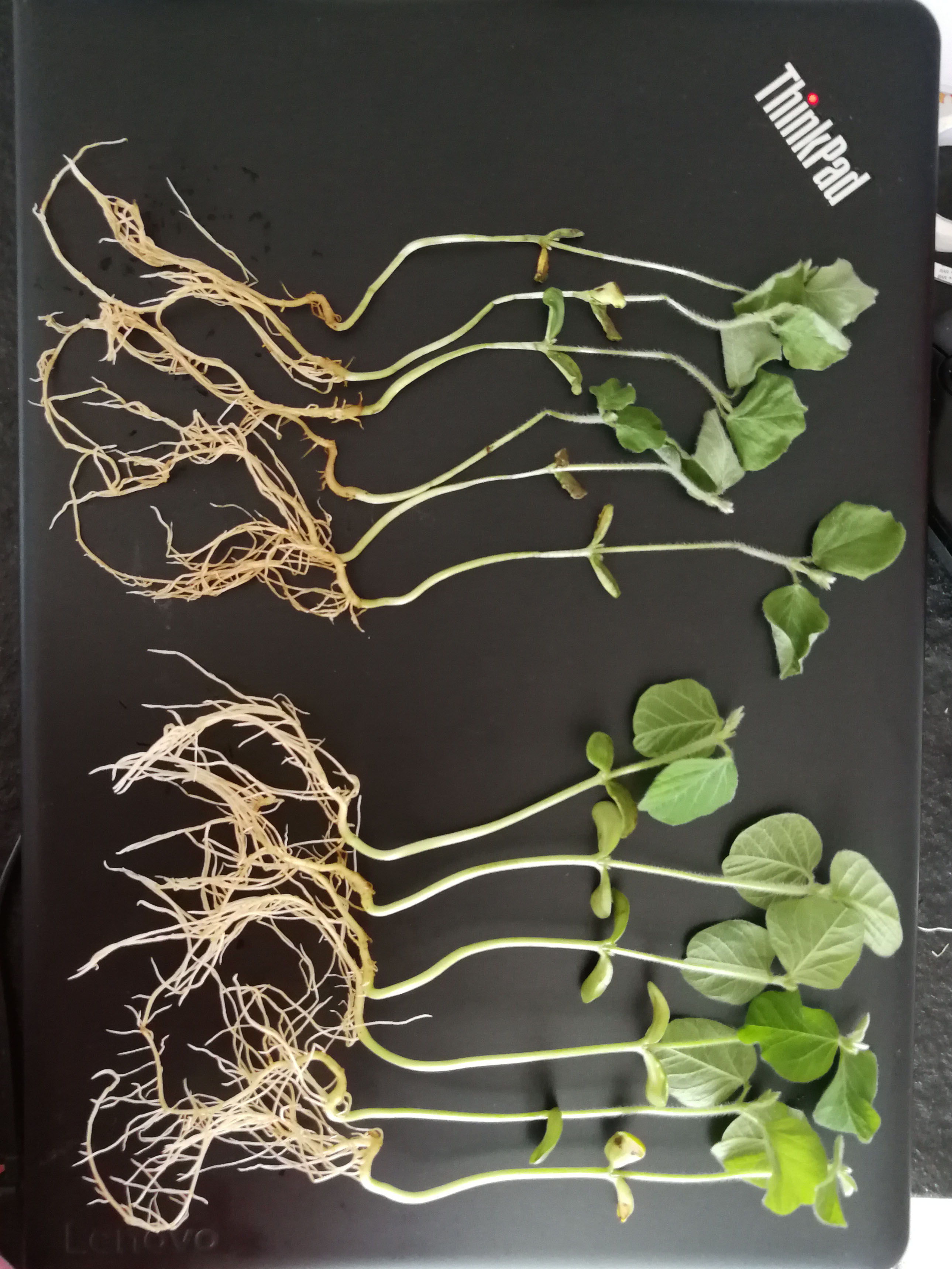

Supplement: S1 Data — (ZIP) [file pone.0267502.s010.zip › Figure 6A-2.jpg]

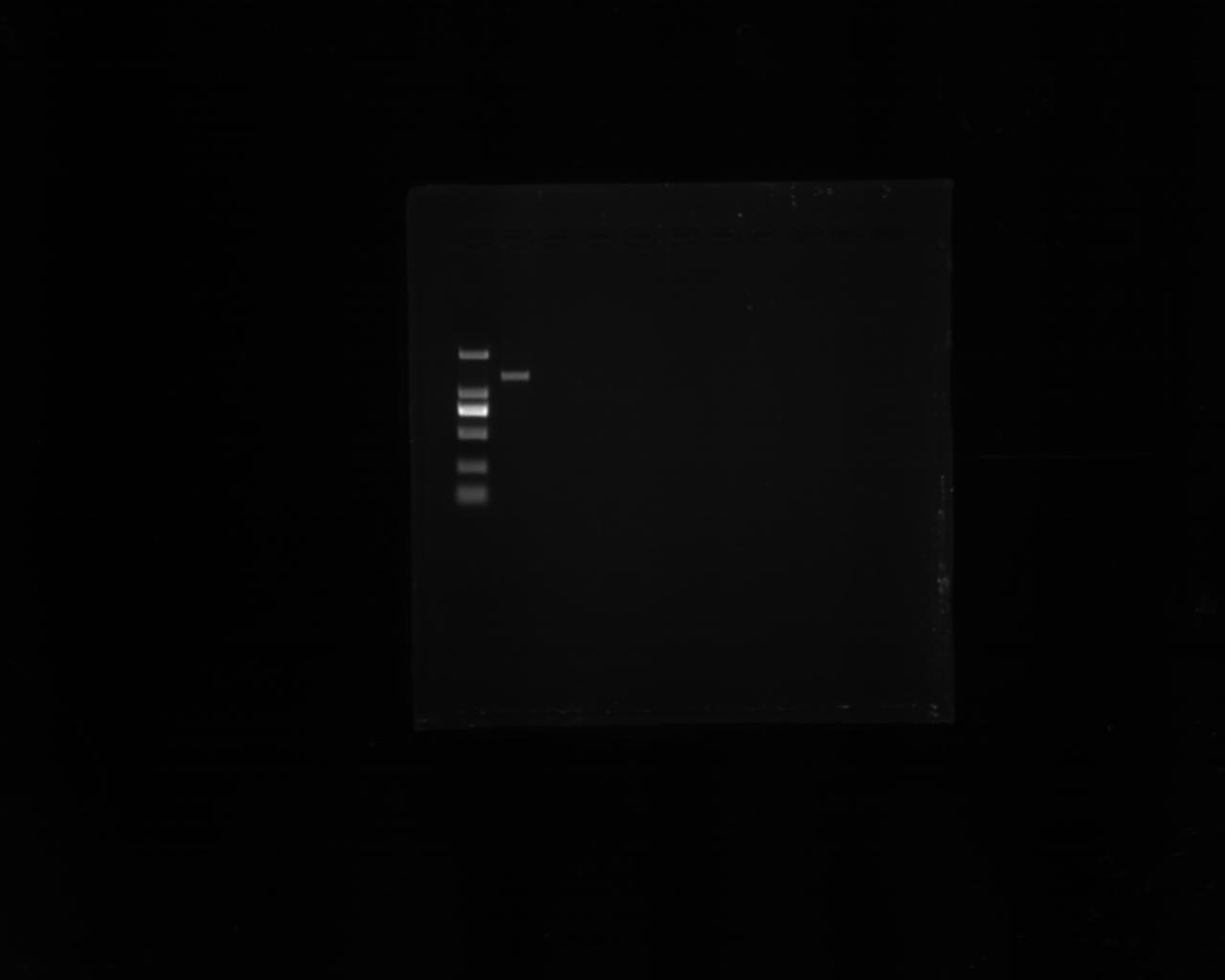

Supplement: S1 Data — (ZIP) [file pone.0267502.s010.zip › Figure S1.jpg]

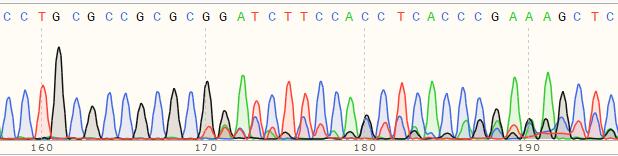

Supplement: S1 Data — (ZIP) [file pone.0267502.s010.zip › Supplementary Figure 4-GmTCP19L-SP1 (#01).jpg]

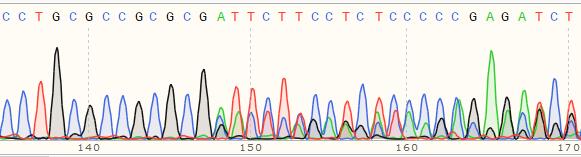

Supplement: S1 Data — (ZIP) [file pone.0267502.s010.zip › Supplementary Figure 4-GmTCP19L-SP1 (#02).jpg]

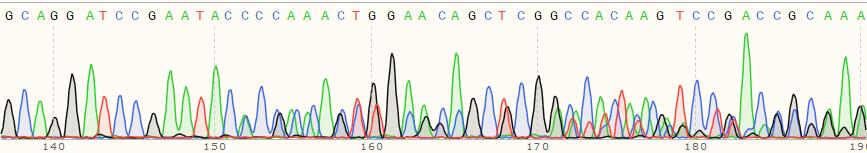

Supplement: S1 Data — (ZIP) [file pone.0267502.s010.zip › Supplementary Figure 4-GmTCP19L-SP1 (#07).jpg]

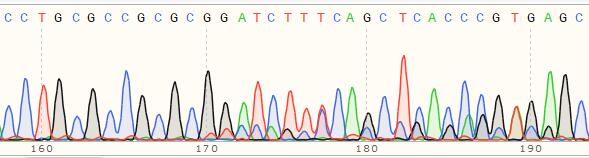

Supplement: S1 Data — (ZIP) [file pone.0267502.s010.zip › Supplementary Figure 4-GmTCP19L-SP1 (#08).jpg]

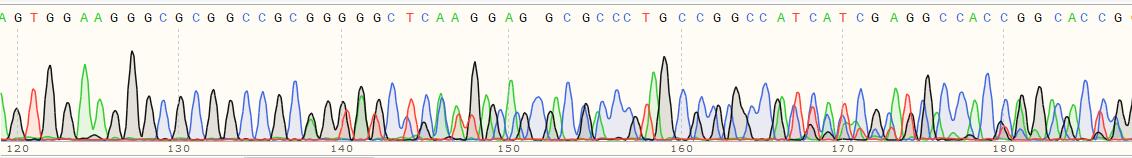

Supplement: S1 Data — (ZIP) [file pone.0267502.s010.zip › Supplementary Figure 4-GmTCP19L-SP1 (#10).jpg]

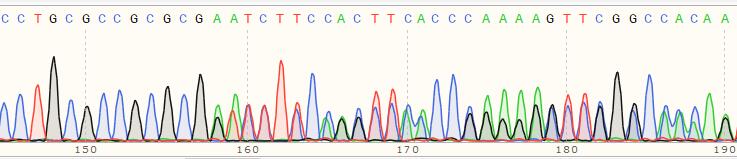

Supplement: S1 Data — (ZIP) [file pone.0267502.s010.zip › Supplementary Figure 4-GmTCP19L-SP1 (#20).jpg]

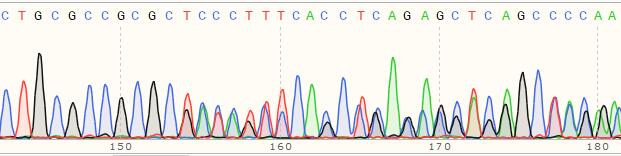

Supplement: S1 Data — (ZIP) [file pone.0267502.s010.zip › Supplementary Figure 4-GmTCP19L-SP1 (#21).jpg]

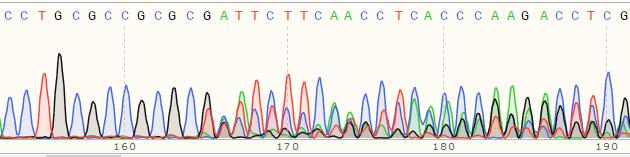

Supplement: S1 Data — (ZIP) [file pone.0267502.s010.zip › Supplementary Figure 4-GmTCP19L-SP1 (#51).jpg]

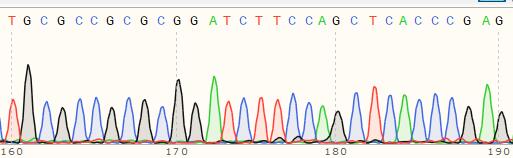

Supplement: S1 Data — (ZIP) [file pone.0267502.s010.zip › Supplementary Figure 4-GmTCP19L-SP1(WT).jpg]

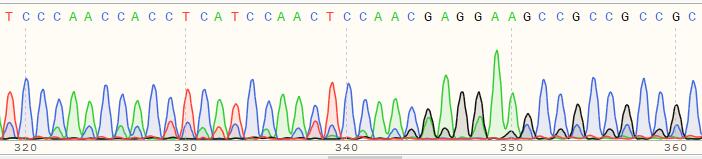

Supplement: S1 Data — (ZIP) [file pone.0267502.s010.zip › Supplementary Figure 4-GmTCP19L-SP2 (#02).jpg]

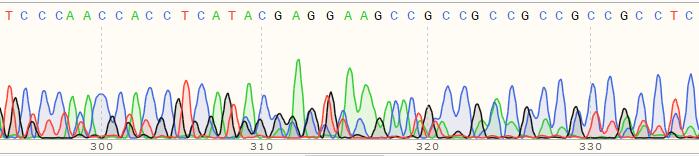

Supplement: S1 Data — (ZIP) [file pone.0267502.s010.zip › Supplementary Figure 4-GmTCP19L-SP2 (#07).jpg]

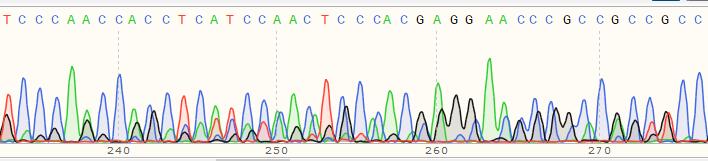

Supplement: S1 Data — (ZIP) [file pone.0267502.s010.zip › Supplementary Figure 4-GmTCP19L-SP2 (#10).jpg]

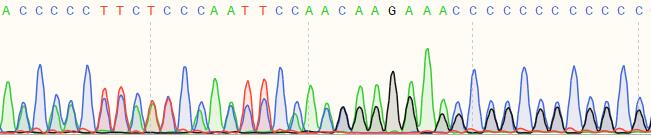

Supplement: S1 Data — (ZIP) [file pone.0267502.s010.zip › Supplementary Figure 4-GmTCP19L-SP2 (#20).jpg]

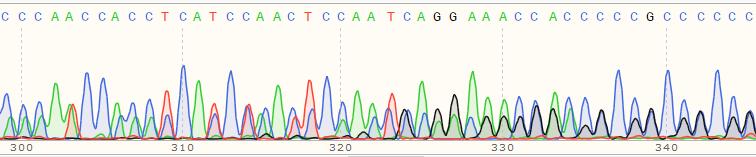

Supplement: S1 Data — (ZIP) [file pone.0267502.s010.zip › Supplementary Figure 4-GmTCP19L-SP2 (#21).jpg]

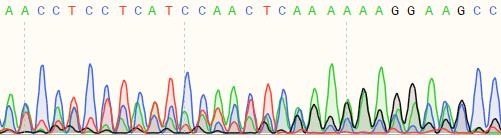

Supplement: S1 Data — (ZIP) [file pone.0267502.s010.zip › Supplementary Figure 4-GmTCP19L-SP2 (#51).jpg]

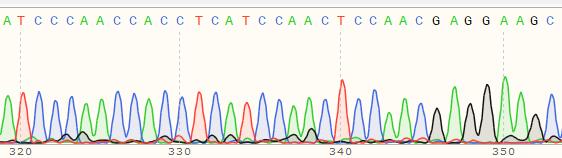

Supplement: S1 Data — (ZIP) [file pone.0267502.s010.zip › Supplementary Figure 4-GmTCP19L-SP2(WT).jpg]
