# Supplementary material for: CRISPR/Cas9-mediated targeted mutagenesis of GmTCP19L increasing susceptibility to Phytophthora sojae in soybean
Source: PLoS One. 2022 Jun 9;17(6):e0267502. doi: 10.1371/journal.pone.0267502 (PMC9182224; doi:10.1371/journal.pone.0267502)
Supplement: S1 File — (DOCX) [file pone.0267502.s011.docx]

**S1 Fig. The sequences of the Cas9 and *Glycine max U6* promoter used in the present study.**

**S2 Fig.** **The basic architecture of the constructs used for** ***GmTCP19L*-CRISPR/Cas9-mediated genome editing.** GmU6, *Glycine max U6* promoter. sgRNA, small guide-RNA. *GmTCP19L*-SP1/SP2, two target sites in the exon of *GmTCP19L*. Cas9-F/R, the primers of the detection region for Cas9. NLS, nuclear localization sequence. The *bar* gene driven by a CaMV 35S promoter is used as a screening marker. Kan, kanamycin resistance gene. pVS1, pVS1 replication origin. STA, pVS1 stability function.

**S3 Fig. Phylogenetic tree analysis of GmTCP19L with the 24 TCP transcription factor members of Arabidopsis. (A)** The PCR amplified products of *GmTCP19L*. M, DL2000 DNA Maker. **(B)** Phylogenetic tree analysis of GmTCP19L with the 24 TCP transcription factor members of Arabidopsis.

**S4 Fig.** **Heterozygous targeted mutagenesis of *GmTCP19L* induced by CRISPR/Cas9 in the T0 generation.** WT, wild-type soybean plant.

**S5 Fig. Amino acid sequence alignment of *GmTCP19L* mutations with WT.** The TCP-like domain was marked with a black box. Nuclear location signal was marked with a dashed black box.

**S6 Fig. Frameshift mutations at two target sites of *GmTCP19L* generated premature translation termination codons.** CDS, coding sequence. Blue capital letter, target sequence. Red capital letter, protospacer adjacent motif. Dashes, deletions. Yellow rectangle, termination codon.

**S1 Table. Primer** **sequences used in the present study.**

| Primer name | Primer sequence | Purpose |
| --- | --- | --- |
| *TCP19L*-F | ATGGATCAAGACGACGACGA | To amplify the CDS regions of *GmTCP19L* |
| *TCP19L*-R | ACTCTGCGCGTGAGTCCC |  |
| *GmTCP19L*-F | GTCCCCAAACAGAACAACGC | To amplify the regions which span the target site |
| *GmTCP19L*-R | TACTCCACCCGAGGGGTTAG |  |
| *OFF1*-F | GGCGTTTCCGCTTCTTGT | To examine potential off-target site 1 |
| *OFF1*-R | AACCGACCTCAGCACCAG |  |
| *OFF2*-F | CATAATGAGCCGAAAACTGC | To examine potential off-target site 2 |
| *OFF2*-R | TGGAAAACCCATTGAAACC |  |
| *OFF3*-F | TGGCTTTTGTTTGAGGGTG | To examine potential off-target site 3 |
| *OFF3*-R | GGCTCTTGACTTTTCTTAGGCT |  |
| *OFF4*-F | CCAAAATCAAGGACCAAAAC | To examine potential off-target site 4 |
| *OFF4*-R | AAATGTAATCCATCAAGACCAAG |  |
| *Cas9*-F | CTCCCGGATGAACACTAAGTAC | To amplify a part of the Cas9 coding sequence |
| *Cas9*-R | CAGGGTAATCTCGGTCTTGAAA |  |
| *PsACT*-F | ACTGCACCTTCCAGACCATC | *P. sojae* *actin* gene used for qPCR measurement of pathogen levels |
| *PsACT*-R | CCACCACCTTGATCTTCATG |  |
| *GmCYP2*-F | CCCCTCCACTACAAAGGCTCG | *G. max* *CYP2* gene used for qPCR measurement of pathogen levels |
| *GmCYP2*-R | CGGGACCAGTGTGCTTCTTCA |  |
| *GmActin*-F | CGGTGGTTCTATCTTGGCATC | as qRT-PCR reference |
| *GmActin*-R | GTCTTTCGCTTCAATAACCCTA |  |

**S2 Table. Potential off-target analysis at the two target sites of *GmTCP19L* in the T1 generation.**

| Target site | Putative off-target site | | | | No. of plans sequenced^c^ | No. of plants with mutations |
| --- | --- | --- | --- | --- | --- | --- |
|  | Gene locus | Region | Sequence^a^ | MMs^b^ |  |  |
| *GmTCP19L*  -SP1 | Glyma17g14160  17:-10922684 | exon | GGTcAGCTGGAAGATCCGgGCGG | 2 | 29 | 0 |
|  | Glyma10g17850  10:-22202782 | exon | GGgGcGCTtGAAGATCCGCaAGG | 4 | 29 | 0 |
| *GmTCP19L*  -SP2 | Glyma04g15120 4:-15495238 | exon | CACCTCAgCaAACTCCAACtGGG | 3 | 15 | 0 |
|  | Glyma05g24520  5:-30684999 | intron | aACCTCATCCAAgTCCAAgGTAG | 3 | 15 | 0 |

a Mismatched bases are shown in lowercase letters.

b No. of mismatched bases.

c T1 plants identified as biallelic mutants of *GmTCP19L*.
