# Supplementary material for: CRISPR/Cas9-mediated targeted mutagenesis of GmTCP19L increasing susceptibility to Phytophthora sojae in soybean
Source: PLoS One. 2022 Jun 9;17(6):e0267502. doi: 10.1371/journal.pone.0267502 (PMC9182224; doi:10.1371/journal.pone.0267502)
Supplement: Attachment — Submitted filename: Response to Reviewer#3.docx [file pone.0267502.s012.docx]

Dear Reviewer,

We appreciate very much the valuable suggestions from you. We have carefully considered every query and given relevant explanation or correction titled “CRISPR/Cas9-mediated targeted mutagenesis of *GmTCP19L* increasing susceptibility to *Phytophthora sojae* in soybean”.

The explanations or corrections are as following:

Q1: First, there is no data presented to support the claim that there were no off-target effects from their CRISPR/Cas9 activity. The authors claim there are no off-target effects, yet only provide a supplementary table listing “number of plants with mutations” in off target sites, filled with zeros. The authors describe using the CRISPR-P analysis program, which provides scores based on strength of sequence alignment with a CRISPR construct. The authors ought to describe what threshold they used to identify putative “off-target” candidates, and they also ought to provide some chromatograms to show that these putative off-target sites were not mutated by the CRISPR constructs.

We have provided some chromatograms in Figure 3 to show that the putative off-target sites were not mutated by the CRISPR constructs in the revised manuscript.

Q2: Second, I don’t think the authors explore enough phenotypes of their mutants. The authors acknowledge at many points in the manuscript that this class of transcription factor play a wide range of roles in growth, development, flowering, circadian rhythm, etc. But, the authors only present one phenotype – susceptibility to 1 isolate of *P. sojae*. Therefore, the increased susceptibility to *P. sojae* could be one of many phenotypes of these mutant lines, which could compromise the conclusion that *GmTRP19* has a direct role in resistance to *P. sojae*. Additionally, the phenotype that the authors do test are only observational, with no numeric data or statistics to suppor the conclusion of increased susceptibility in the mutants.

We are very grateful to you for your valuable suggestions. In previous studies, there were reports that a few of TCP genes also play a wide range of roles in leaf development (Efroni et al, 2008), leaf morphogenesis (Palatnik et al., 2003), leaf senescence (Schommer et al., 2008), trichome development (Steiner et al., 2012; Vadde et al., 2018) flower development (Nag et al., 2009; Koyama et al., 2011; Li et al., 2021) and circadian clock (Giraud et al., 2010). So, in the follow-up work, it is valuable that exploring enough phenotypes of the mutants, and it will improve the interpretation of the findings that the function of the *GmTCP19L* response to *P*. *sojae* infection. In the revised manuscript, we supplemented this relevant content in the discussion section.

Q3: Specific comments:

1. Page 2, Line 15: Remove “the” before “conserved domains”

We have removed the word “the”.

2. Page 4, Line 3: What is the FT-FD complex. Please list in full before using an abbreviation. Page 4, Line 4: What is the AP1 gene, and what is the relevance of AtTCP12 and AtTCP18 binding to it?

We have rephrased this sentences as “AtTCP12/AtTCP18 can integrate into the FLOWERING LOCUS T (FT)‒FD complex to control floral initiation and also directly bind the promoter of downstream floral meristem identity gene *APETALA1*(*AP1*) to enhance its transcription and regulate flowering”.

3. Page 4, Line 17: Hyaloperonospora arabidopsidis is not a gram-negative bacteria. It is an oomycete. Please correct.

We have corrected it.

4. Page 5, Line 9: Remove “related to *P. sojae*”. You describe the connection between the gene and *P. sojae* later in the sentence.

We have removed the words “related to *P. sojae*”.

5. Page 5, Line 18: Remove “the” before “resistance”. There is no singular resistance to *P. sojae* – there is single, R-gene mediated resistance, and partial resistance (Dorrance, 2018 doi.org/10.1080/07060661.2018.1445127). The GmTPS19 gene is not an R-gene.

We have removed the word “the” before “resistance”.

6. Page 6, line 6: Williams 82 has high partial resistance to *P. sojae*, but only 1 R-gene mediating robust resistance to specific pathotypes of *P. sojae* (Dorrance, 2018).

In previous studies, there were reports that a few of genes were induced by *P*. *sojae*, such as N-rich protein (Ludwig and Tenhaken, 2001), *GmPR10* (Xu *et al*., 2014), *GmPIB1* (Dong *et al*., 2018) and *GmBTB* (Zhang et al., 2021). Thus, we speculate that there maybe *P*. *sojae* induced genes changed in their expression when the expression of *GmTCP19L* is modified, which might affect the resistance of soybean response to *P. sojae* infection*.* So, in the follow-up work, it is valuable that making a deeper research on protein interaction and regulatory network building. In the revised manuscript, we supplemented this relevant content in the discussion section.

7. Page 6, line 11: Capitalize “Professor Zhang”.

We have replaced “professor” with “Professor”.

8. Page 8, line 6: What is a PAT/Bar “test strip”? Did you spray these plants with gufosinate? Did you do PCR to detect the presence / absence of this gene? Did you get strips from a company? If so, where? More detail needed here for reproducibility.

We have rephrased this sentences as “PAT/Bar test strip (Catalog No. STX 14200, Agdia, USA) was used to identify the selective marker *bar* gene”.

9. Page 8, line 12: “Two off-target sites for each guide would be selected” does this mean there were 2 off target sites detected by CRISPR-P analysis? Or only 2 were selected for analysis of off-target effects? There’s a big difference, please clarify.

Page 12, lines 8-9: How “likely” were these two off-target sites predicted to be affected? How many other off-target sites were identified at a lower “likelihood”? Can you provide the results of the CRISPR-P analysis in the supplemental files, or list here in some form?

I'm sorry that there is not appropriate in our description here. And the most potential off-target analysis at the two target sites of *GmTCP19L* in the T1 generation have been listed in S2 Table. Meantime, we have provided some chromatograms in Figure 3 to show that the putative off-target sites were not mutated by the CRISPR constructs in the revised manuscript.

10. Page 10, lines 7-8: Please clarify the language here a bit. You list two numbers and use “respectively”, but don’t list both conditions. Please change to something like this “…had heterozygous mutations at the first (SP1) and second (SP2) target sites, respectively (S3 Fig in S1 File)”.

We have checked and corrected this problem.

11. Page 10, line 16: Please don’t use “PTCs” as an abbreviation. This is unnecessary.

We have removed the abbreviation “PTCs”.

12. Page 12, lines 12-13: “no mutations were observed” yet the data to support this is just a table with 0’s listed. Please provide the chromatograms to show evidence that there were no off-target effects on DNA sequence.

We have provided some chromatograms in Figure 3 to show that the putative off-target sites were not mutated by the CRISPR constructs in the revised manuscript.

13. Page 13, Line 17: “all of their…” all of their what? I think there is a word missing here. Or perhaps “their” is meant to be “them”?

We have replaced “their” with “them”.

14. Figure 4: These soybeans do not look like they are growing in vermiculite, as described throughout the manuscript. Explain.

In low-generation, the number of homozygous plants obtained by screening is small, and one of the most important purposes of this research is to obtain new germplasm materials for breeding research, in this case, in order to improve survival rate of the mutant plants, seeds collected from the T0 generation were sown in pots filled with sterile vermiculite. In the T2 generation, for disease resistance analysis, seeds collected from T1 ‘transgene-free’ mutants were sown in pots filled with nutritious soil and grown in a greenhouse. So, there is an error in our description here, and we have checked and corrected the description in the revised manuscript.

15. Page 15, lines 15-19: This brings up a great point. Did any of your *GmTRP19* mutants display any other phenotypes besides increased sensitivity to *P. sojae*? Were there any developmental phenotypes (delayed germinating, effects on plant height, flowering, etc.)?

We are very grateful to you for your valuable suggestions. In previous studies, there were reports that a few of TCP genes also play a wide range of roles in leaf development (Efroni et al, 2008), leaf morphogenesis (Palatnik et al., 2003), leaf senescence (Schommer et al., 2008), trichome development (Steiner et al., 2012; Vadde et al., 2018) flower development (Nag et al., 2009; Koyama et al., 2011; Li et al., 2021) and circadian clock (Giraud et al., 2010). So, in the follow-up work, it is valuable that exploring enough phenotypes of the mutants, and it will improve the interpretation of the findings that the function of the *GmTCP19L* response to *P*. *sojae* infection. In the revised manuscript, we supplemented this relevant content in the discussion section.

16. Page 16, line 4: What makes *GmTRP19* a “new” member of this family?

We have rephrased this sentences as “*GmTCP19L* is the first *TCP* gene was identified in soybean response to *P. sojae* infection:.

17. Page 16, lines 5-9: This entire sentence is an incomplete sentence. Authors could remove “Having demonstrated that”, to make it complete.

We have rephrased this sentences as “In previous studies, TCP transcription factors have been shown to be play a versatile regulatory role at the different stage of plant growth and development”.

18. “We obtained a novel type of mutations”

We have rephrased this sentences as “We obtained the transgene-free mutants with 2-bp deletion at *GmTCP19L* coding region, and the frameshift mutations produced premature translation termination codons and truncated GmTCP19L proteins, increasing susceptibility to *P. sojae* infection in the T2-generation”.

19. “which suggesting”

We have removed the word “which”.

20. “this gene resistance to *P. sojae*”

We have rephrased this sentences as “studied the function of this gene responses to *P. sojae* infection”.

21. “exhibited more sensitive to *P. sojae*”

We have rephrased this sentences as “In T2-generation, the homozygous mutants for null alleles of *GmTCP19L* frameshift mutated by a 2-bp deletion without any transgenic element resulted in enhanced susceptibility to *P. sojae* infection by decreasing the activity of antioxidant defense system”.

22. “All of three types”

We have rephrased this sentences as “The frameshift mutations of three types at two target sites of *GmTCP19L*”.

23. “an important food and economic crops”

We have rephrased this sentences as “Soybean [*Glycine max* (L.) Merr.] is an important food crop with great economic value that abundant protein and oil”.

I hope the revision is satisfactory and please contact me shall you have further questions.

Yours sincerely,

Sujie Fan

E-mail: fansujie2009@163.com
