# Supplementary material for: CRISPR/Cas9-mediated targeted mutagenesis of GmTCP19L increasing susceptibility to Phytophthora sojae in soybean
Source: PLoS One. 2022 Jun 9;17(6):e0267502. doi: 10.1371/journal.pone.0267502 (PMC9182224; doi:10.1371/journal.pone.0267502)
Supplement: Attachment — Submitted filename: Response to Editor.docx [file pone.0267502.s013.docx]

Dear editor,

We appreciate very much the valuable suggestions from you. We have carefully considered every query and given relevant explanation or correction titled “CRISPR/Cas9-mediated targeted mutagenesis of *GmTCP19L* increasing susceptibility to *Phytophthora sojae* in soybean”. And the explanations or corrections are as following:

1. Page 2 line 1. Delete (*P. sojae*)

We have removed the abbreviation “*P. sojae*”.

 2. Page 2 line 4. The Abstract should be revised. If the authors already known that *GmTCP19L* controlling soybean resistance to *P. sojae*, there will be no need for the rest of study presented in the manuscript. The sentence here should be the background, not the conclusion. Description such as “and generated targeted mutants of *GmTCP19L* gene, which was previously related to involve in soybean responses to *P. sojae*” would make more sense.

We have rephrased this sentences as “*Agrobacterium*-mediated transformation was used to introduce the CRISPR/Cas9 expression vector into soybean cultivar ‘Williams 82’ and generated targeted mutants of *GmTCP19L* gene, which was previously related to involve in soybean responses to *P. sojae*”.

 3. Page 2 line 10. The authors emphasized multiple times throughout the manuscript regarding the mutants without "any transgenic elements." This is a bold statement as the authors only detect BAR and a PCR fragment of Cas9 vector. In other words, if there is any other part of vector elements remained in the mutant genome, the methods will have no capability of detecting them. Therefore, I suggest the authors to revise these statements in a conserved way, e.g. the BAR and Cas9 sequences were undetectable using strip and PCR methods, respectively. Others: Page 6 line 1, Page 9 line 6, Page 10 line 12, Page 14 line 15 (Figure 4 title), Page 15 line 6, Page 20 line 12 etc.

We appreciate very much the valuable suggestions from you, and we have rephrased this sentences as “The new soybean germplasm with homozygous *tcp19l* mutations but the BAR and Cas9 sequences were undetectable using strip and PCR methods, respectively” or “mutants without the BAR and Cas9 sequences of the CRISPR/Cas9 vector”.

4. Page 2 line 14. TCP should be spell-out for clarity especially in the every beginning of the text.

TCP has been spelled out clearly in the beginning of the text as you suggested.

5. Page 3 line 2. Is “shown” to

We have replaced “thought” with “shown”.

6. Page 3 line 4. classified “into” Class I

We have added the word “into” as you suggested.

 7. Page 3 line 9. Italize cis-

We have made the correction based on your suggestion.

8. Page 3 line 15. Have been found, “and” most of which

We have added the word “and” as you suggested.

 9. Page 3 line 18 to Page 4 line 3. This is a long sentence with many information. Please simplify or separate it.

We have rephrased this sentences as “AtTCP14 and AtTCP15 can modulate cell proliferation in the developing leaf blade and specific floral tissues[26], and also participate in the induction of genes involved in gibberellin biosynthesis and cell expansion by high temperature functionally [27, 28]”.

10. Page 4 line 14. No need for (BR) because it is not mentioned later on.

We have removed the abbreviation “(BR)”.

11. Page 4 line 19 to Page 5 line 1. Please double confirm the meaning of this sentence. It reads that two pathogens target TCP to enhance disease resistance…Would the pathogens do these functions against themselves?

We have checked and corrected the sentences as “AtTCP13, AtTCP15 and AtTCP19 can be targeted by effectors from the gram-negative bacteria *Pseudomonas syringae* and the oomycete *Hyaloperonospora arabidopsidis*, and the plants mutated in *AtTCP13*, *AtTCP15* or *AtTCP19* exhibit altered infection phenotypes”.

12. Page 5 line 10. The authors used the W82a1v1 names throughout the manuscript. However, the popular and common names of soybean genes are already based on the W82a2v1 version, or even a4v1 version. Please revise the name or at least provide W82a2v1 names side-by-side. Others: Page 7 line 2, Figure 3 etc. Please also confirm the name used in the phylogentic tree, it seems to be the a2v1 name.

We have checked and corrected the name as Glyma.05G050400.1 based on the W82a2v1 version.

13. Page 5 line 14. Arabidopsis, “and” this

We have replaced “And then” with “and”.

14. Page 5 line 16. In “the” soybean cultivar

We have added the word “the”.

15. Page 5 line 18. “with” short deletions

We have replaced “by” with “with”.

16. Page 6 line 4. soybean resistance to *P. sojae*.

We have rephrased this sentences as “*GmTCP19L* directly or indirectly regulates soybean resistance to *P. sojae*”.

17. Page 6 line 10. “high resistance to *P. sojae*” is not a specific or scientific description. How high is high? Please be specific, e.g. W82 carries Rps-1k? or what do the authors mean for high?

We have rephrased this sentences as “Williams 82, a *P. sojae*-resistant soybean cultivar carrying resistance gene *Rps1k*, was used for transformation in this study”.

 18. Page 7 line 9-12. Please provide the vector plot with clear locations of primers, the BAR resistance gene, the cloning regions for *GmTCP19L*, and the detection region for Cas9.

The vector plot with clear locations of target sites of *GmTCP19L*, the primers of the detection region for Cas9, and the BAR resistance gene was shown in S2 Fig.

19. Page 7 line 15. More details needed for tri-parental mating.

Suggested by Reviewer, the method of plasmid delivery by tri‐parental mating were specified in the M&M section of the revised manuscript.

20. Page 8 line 8-9. Delete. Frameshift mutation is clear enough.

We have removed the parenthesis “(not multiples of three)”.

 21. Page 8 line 10-13. Please specify the length or region of BAR detection and Cas9 amplicon.

The BAR detection and the region of Cas9 amplicon were specified in the M&M section, we have rephrased this sentences as “PAT/Bar test strip (Catalog No. STX 14200, Agdia, USA) was used to identify the BAR protein following the manufacturer’s instruction, and the primers Cas9-F/R were used to amplify the fragment (349 bp) of the Cas9 (S2 Fig in S1 File)”..

22. Page 9 line 17-18. More details of the methods are needed. How as the *P. sojae* quantified? Disease severity? Counting sporangia? qPCR? In addition, what was the statistical method?

Thanks very much for your valuable comments. There was not a detailed description for pathogen level analysis, and we added the detailed description in the revised manuscript. The relative accumulation of *P.sojae* in infected cotyledons and roots were also analyzed by qPCR using One Step RT-PCR Kit (Code No.:PCR-311, TOYOBO, Japan) on a QuantStudio 3 instrument (Thermo, United States). The expression of the *P. sojae* housekeeping gene *PsACT* (GenBank accession no. XM_009530461.1) relative to the soybean housekeeping gene *GmCYP2* (Glyma.12G024700) (ΔCt = Ct_HK of_ *_P. sojae_* - Ct_HK of soybean_) was calculated. The housekeeping genes of *P. sojae* and soybean were chosen as described previously by Wang *et al.* (2011) , and the primers were listed in S1 Table (in S1 File).

 23. Page 10 line 4. One gram

We have replaced “one gram” with “1 g”.

24. Page 10 line 7. Which part of results were analyzed using “multiple pairwise comparison”? In addition, was the assumption of t test checked? Please specify.

I'm sorry that there is an error in our description here, and we have checked and corrected the description in the revised manuscript. Relative enzyme activity, i.e. the ratio of SOD activity or POD activity in infected roots under *P. sojae* zoospores in *tcp19l* mutants versus non-transformed seedlings at the same time point were measured. Three biological replicates, each with three technical replicates, were averaged and statistically analysed using Student's *t*-test (**P*<0.05, ***P*<0.01). Bars indicate the standard deviation of the mean.

25. Page 11 line 20. Replace “. Simultaneously” to “; meanwhile,”

We have replaced “Simultaneously” to “Meanwhile”.

26. Page 14 line 1. Please provide figure legend for Figure 3. Please verify the version of soybean gene name.

We have supplemented the figure legend in Figure 3 and corrected the soybean gene name based on the W82a2v1 version.

27. Page 15 line 5. In response to

We have rephrased this sentences as “Phenotypes of the mutants in response to *P. sojae* infection”. We have replaced “response to” to “in response to”.

28. Page 15 line 6-7. Are these two T1 lines, *tcp19l*-SP1-T1 7 (2-bp deletion), *tcp19l*-SP1-T2), identical to “*tcp19l*-SP1-T1#02.03 and *tcp19l*-SP1-T1#02.08”? Or which is which? Please clarify.

We have rephrased this sentences as “In the T2 generation, the progeny of T1 homozygous *tcp19l* mutants *tcp19l*-SP1-T1#02.03 and *tcp19l*-SP1-T1#02.08 were grown under natural conditions. Because of the mutant of them were consistent, we number them uniformly as *tcp19l*-SP1-T2, and all of them were homozygous *tcp19l* mutants without the BAR and Cas9 sequences (Table 2)”.

29. Page 16 line -2. Methods for qualification needed.

There was not a detailed description for pathogen level analysis, and we added the detailed description in the M&M section of the revised manuscript. The relative accumulation of *P.sojae* in infected cotyledons and roots were also analyzed by qPCR using One Step RT-PCR Kit (Code No.:PCR-311, TOYOBO, Japan) on a QuantStudio 3 instrument (Thermo, United States). The expression of the *P. sojae* housekeeping gene *PsACT* (GenBank accession no. XM_009530461.1) relative to the soybean housekeeping gene *GmCYP2* (Glyma.12G024700) (ΔCt = Ct_HK of_ *_P. sojae_* - Ct_HK of soybean_) was calculated. The housekeeping genes of *P. sojae* and soybean were chosen as described previously by Wang *et al.* (2011) , and the primers were listed in S1 Table (in S1 File).

30. Page 16 line 9. Please provide figure legend for Figure 5.

We have supplemented the figure legend in Figure 5.

31. Page 16 line 15 to Page 17 line 3. If this is the quantification method, please move to M&M, and specify the primers and provide methods e.g. construction of standard curve? for details.

We have provided the detailed description for pathogen level analysis in the M&M section of the revised manuscript. The relative accumulation of *P.sojae* in infected cotyledons and roots were also analyzed by qPCR using One Step RT-PCR Kit (Code No.:PCR-311, TOYOBO, Japan) on a QuantStudio 3 instrument (Thermo, United States). The expression of the *P. sojae* housekeeping gene *PsACT* (GenBank accession no. XM_009530461.1) relative to the soybean housekeeping gene *GmCYP2* (Glyma.12G024700) (ΔCt = Ct_HK of_ *_P. sojae_* - Ct_HK of soybean_) was calculated. The housekeeping genes of *P. sojae* and soybean were chosen as described previously by Wang *et al.* (2011), and the primers were listed in S1 Table (in S1 File).

32. Page 18 line 12. RNA-Seq

We have replaced “RNA-seq” with “RNA-Seq”

33. Page 19 line 20 to Page 20 line 1. Please provide previse literature for supporting ROS involves in *P. sojae* resistance.

GmPIB1, a bHLH-type transcription factor, has been shown to facilitate resistance to *P. sojae* in *Glycine max* by affecting ROS levels. So, we provided this previse literature for supporting ROS involves in *P. sojae* resistance in the revised manuscript.

34. Page 20 line 11. Delete Now.

We have removed the word “Now”.

35. In Figure 5 and 6, please clarify which lineage does the *tcp19l*-SP1-T2 and *tcp19l*-SP1-T3 from? Are they 2-bp or 1-bp deletion mutants? Are they progenies of *tcp19l*-SP1-T1#02.03 and *tcp19l*-SP1-T1#02.08? Have different mutant lines/lineage being tested for resistance? Please note if only one mutant lineage was used in pathogenicity test, the results may not be convincing. If different mutant lineage, e.g. 2-bp and 1-bp, were tested and the results were consistent, the conclusion will be more reliable.

Thanks very much for your valuable suggestions. And I’m sorry to tell you that because of the mutant of T1 *tcp19l* mutants *tcp19l*-SP1-T1#02.03 and *tcp19l*-SP1-T1#02.08 were consistent (2-bp deletion), we number the progeny of them uniformly as *tcp19l*-SP1-T2, and *tcp19l*-SP1-T3 from *tcp19l*-SP1-T2. We missed resistance identification for 1-bp deletion mutants in the T2 generation because of we eliminated them when screening “transgene-free” mutants. But, in the T1 generation, three types of *tcp19l* mutants (1-bp deletion and 2-bp deletion at target site *GmTCP19L*-SP1, 14-bp deletion at target site *GmTCP19L*-SP2) were detected by sequence alignment with the WT sequence. At that time, we considered that the number of *tcp19l* mutants is small, and one of the most important purposes of this research is to obtain new germplasm materials for breeding research, so, in order to avoid death of *tcp19l* mutants after *P. sojae* infection, the living cotyledons of three types of *tcp19l* mutants were selected to investigate resistance to *P. sojae*. After 3 days of incubation with zoospores of *P. sojae*, a remarkable difference in the development of disease symptoms was observed. In *tcp19l* mutants, the cotyledons became soft and exhibited clear water-soaked lesions compared with those of the WT, and the relative biomass of *P. sojae* in infected living cotyledons was significantly (P<0.05) higher in *tcp19l* mutants (1-bp deletion and 2-bp deletion) than that in WT. Because of we did not report these results in previous manuscript, we supplemented this relevant content in the revised manuscript.

36. In addition to these editorial suggestions, the reviewers would like to know more about the phenotypes of 2-bp or 1-bp deletion mutants, and/or *tcp19l*-SP1-T1#02.03 and *tcp19l*-SP1-T1#02.08. The reviewers’ opinion is highly respected because if there are other phenotypic defects, the damping-off may be a side-effect due to other genetic problems instead of purely disease susceptibility. Your feedback in the discussion included several literatures mostly based on Arabidopsis (Page 18 line 16-20), but not direct observation on your T0, T1, T2, or T3 soybean mutants. Therefore, I would like to invite you to provide more details for the final editorial and content improvement.

We are very grateful to you for your valuable suggestions. In the low generation, the number of *tcp19l* mutants is small, and one of the most important purposes of this research is to obtain new germplasm materials for breeding research, so, we missed more phenotypes identification for mutants. But, we identified the off-target sites in the progeny of *tcp19l* mutants and no mutations were observed in the examined potential off-target sites, meanwhile, we did not find any other phenotypic changes from T0, T1, T2, or T3 *tcp19l* mutants. In the follow-up work, we will continue to multiply the number of seeds and make a deeper research on the phenotypes of *tcp19l* mutants, such as leaf morphogenesis, leaf senescence, flower development, hormone signaling, and response to other stresses. In the discussion section, we supplemented this relevant content in the revised manuscript.

I hope the revision meets the high standard of PLoS ONE and please feel free to contact me shall you have any questions.

Yours sincerely,

Sujie Fan

E-mail: fansujie2009@163.com
